# Supplementary material for: Assessing the efficacy, safety and utility of closed-loop insulin delivery compared with sensor-augmented pump therapy in very young children with type 1 diabetes (KidsAP02 study): an open-label, multicentre, multinational, randomised cross-over study protocol
Source: BMJ Open. 2021 Feb 12;11(2):e042790. doi: 10.1136/bmjopen-2020-042790 (PMC7883854; doi:10.1136/bmjopen-2020-042790)
Supplement: Supplementary data [file bmjopen-2020-042790supp001.pdf]

Supplementary Material

Supplementary Appendix

1. Training Details Page 2
2. CamAPS FX closed-loop system Page 3
3. Blood sampling schedule Page 4
4. Questionnaires Page 5
5. Composition of study management groups Page 6
6. Example consent form Page 7
7. Statistical analysis plan Page 9
8. Study protocol Page 22

Fuchs J, *et al. BMJ Open* 2021; 11:e042790. doi: 10.1136/bmjopen-2020-042790

Supplementary Material

1. Training details

Pre-randomisation training

Participants and their families will be educated on the use of the study continuous glucose monitoring (CGM) device and the study insulin pump. For CGM training particular attention will be paid to insertion and initiation of a sensor session, when a finger-stick blood glucose (BG) reading needs to be taken, how and when to calibrate successfully, and BG targets and alarm settings. Parents will be trained in handling real-time CGM information including glucose arrow trends and reported high and low glucose. Hypo- and hyperglycaemia alarms can be activated and customised as per participants' requirements.

For study pump training particular attention will be paid to insulin cartridge and infusion set changes as well as the correct priming procedures. Refresher training on the importance and accuracy of carbohydrate counting, insulin-to-carbohydrate ratios, and correction factors will also be provided. Parents will be instructed on the correct use the bolus calculator. The study team will review sick day rules, exercise advice and how to deal with hypo- and hyperglycaemia with the participants.

Post-randomisation training

Closed-loop training

Participants will be trained on connection and disconnection of the closed loop system as well as switching between closed loop and sensor-augmented pump therapy. During the closed-loop period, meal boluses will be delivered by the insulin pump based on carbohydrate estimation. Specific instructions during closed-loop related to exercise management, sick day rules, hypo- and hyperglycaemia management and technical troubleshooting will also be reviewed with the participants by the study team.

Sensor-augmented pump therapy

Participants will receive refresher training including key aspects of insulin pump and CGM use.

Supplementary Material

2. CamAPS FX closed loop system

The CamAPS FX closed loop system comprises:

- Dana Diabecare RS insulin pump (Sooil, Seoul, South Korea)
- Factory calibrated Dexcom G6 real-time CGM sensor (Dexcom, San Diego, CA, USA)
- An Android smartphone hosting CamAPS FX app with the model predictive control algorithm (University of Cambridge, Cambridge, UK) and communicating wirelessly with the insulin pump and CGM transmitter
- Real-time upload capabilities to diabetes management system Diasend (Glooko/Diasend, Göteborg, Sweden).

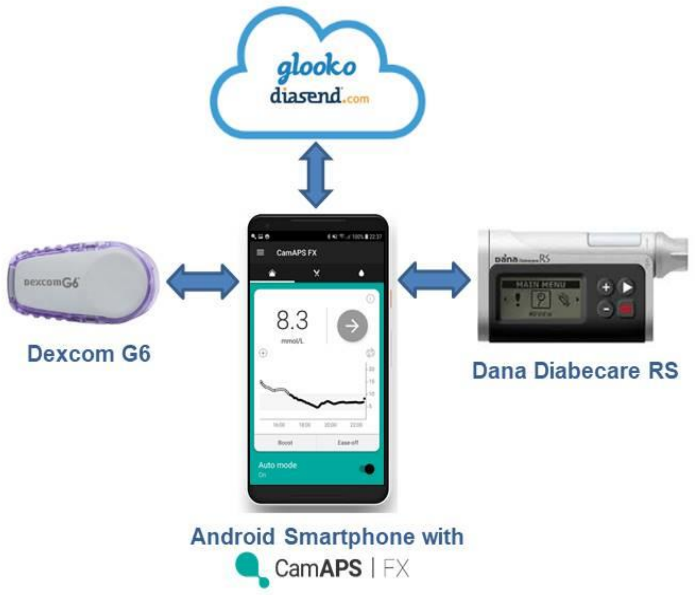

3. Blood sampling schedule

**Visit 1** – recruitment	**Visit 6** – end of 1st arm	**Visit 9** – end of 2nd arm
Local lab HbA1c	Local lab HbA1c	Local lab HbA1c

Supplementary Material

4. Questionnaires

Measure	*Respondent*	*Construct Measured / Relevant Points*	*Timepoint*
**Strengths and Difficulties** Questionnaire (SDQ) (61)	Parents/ Guardians	This is a widely used 25 item self-report inventory behavioural screening questionnaire for children and adolescents. The same 25 items are included in questionnaires for completion by the parents.	Baseline, Visits 6 & 9
**Hypoglycaemia Fear** Survey (HFS) Parents of Young Children (62-64)	Parents/ Guardians	Validated questionnaire to measure several dimensions of fear of hypoglycaemia. It consists of a "Behaviour subscale" that measures behaviours involved in avoidance and over-treatment of hypoglycaemia and a "Worry subscale" that measures anxiety and fear surrounding hypoglycaemia.	Baseline, Visits 6 & 9
**WHO Well-Being Index** (WHO-5) (65)	Parents/ Guardians	Widely used 5-item questionnaire assessing subjective psychological well-being.	Baseline, Visits 6 & 9
**Epworth sleepiness** scale (ESS) (66)	Parents/ Guardians	Simple 8-item questionnaire to measure the parents general level of daytime sleepiness and average sleep propensity in daily life.	Baseline, Visits 6 & 9
Insulin delivery Systems: Perceptions, Ideas, Reflections, and Expectations **(INSPIRE) Survey**	Parents/ Guardians	Measures the psychological side of automated insulin delivery. Child version has 27 items; Parent version has 30 items.	Baseline, Visits 6 & 9
**Pittsburgh Sleep Quality Index** (PSQI)	Parents/ Guardians	The PSQI is a 19 item questionnaire that holistically assesses sleep quality and sleep duration.	Visit 8
**Children's Sleep Habit** Questionnaire (CSHQ)	Parents/ Guardians	The CSHQ is a validated retrospective 45-item questionnaire that examines sleep behaviour in young children.	Visit 8

BMJ Publishing Group Limited (BMJ) disclaims all liability and responsibility arising from any reliance placed on this supplemental material which has been supplied by the author(s)

5. Composition of trial management groups

Data safety and monitoring board (DSMB) will comprise an independent chairperson and two external experts.

Trial steering committee (TSC) will consist of an independent chairperson, a public and patient involvement (PPI) representative, the Chief Investigator, and the leaders of work packages (WP) 2 (pilot study), WP3 (main study) and WP4 (data management) of the KidsAP consortium. The Principal Clinical Investigators may also participate.

Trial management group (TMG) will consist of the Chief Investigator, Study Coordinators, and Study Data Manager. The Principal Clinical Investigators may also participate in the meetings of the TMG.

Fuchs J, *et al. BMJ Open* 2021; 11:e042790. doi: 10.1136/bmjopen-2020-042790

Supplementary Material

6. **Example consent form**

KidsAP02 - The artificial pancreas in very young children with T1D

Institutional logo

Patient ID Label:

Centre NRES number: ....

Patient identification number for this trial: ....

Name of child: ....

CONSENT FORM
(Caregiver for child's participation)

Title of Project: The artificial pancreas in very young children with Type 1 Diabetes (KidsAP02)

Name of Researcher: ....

Please initial box

1. I confirm that I have read and understood the information sheet version .... dated .... for the above study and have had the opportunity to consider the information and I am satisfied with the answers I have been given. ☐
2. I understand that my child's and my participation is voluntary and that we are free to withdraw at any time, without giving any reason, without your child's medical care or legal rights being affected in any way. ☐
3. I agree to my child's participation in the study, and to follow the study team's instructions for the purposes of the study. ☐
4. I give consent for the taking of capillary blood samples from my child. ☐
5. I agree to complete the questionnaires as outlined in the patient information sheet. ☐
6. Qualitative Interviews:

   a. I agree to participate in the interviews. ☐

   b. I agree for my contact details to be given to the interview study team. ☐

   c. I agree for the interview to be digitally recorded. ☐

   d. I understand that anonymised quotes may be used in reports and publications. ☐
7. If I choose to communicate with the research team via email, I acknowledge the associated risks as outlined in the information sheet. ☐

250941_Consent Form for caregivers_V3.0_05 March 2019 Page 1 of 2

Fuchs J, *et al. BMJ Open* 2021; 11:e042790. doi: 10.1136/bmjopen-2020-042790

8. I give consent to my child's GP being informed of his/her participation in this study. ☐

9. I give consent for the collecting of my child's medical data related to the study as outlined in the information sheet. ☐

10. I give consent to the sharing of anonymised research data arising from this study with other study partners, researchers and for publication purposes in Europe and outside Europe. This sharing of data may continue after the research study is completed. ☐

11. I understand that relevant sections of my child's medical notes and data collected during the study may be looked at by individuals from the NHS Trust and regulatory bodies for audit or monitoring purposes. ☐

12. I agree to return the study devices at the end of the study or earlier if consent is withdrawn. ☐

13. I give consent for my child's personal data to be kept by the clinical research team for up to 5 years after termination of the study so that they may contact me in the future with results of this and other similar studies. ☐

14. I give consent for the collecting of my child's medical data related to the study and transfer to a data controller and/or processor located in USA, as outlined in the information sheet. ☐

______________________	______________	______________
Name of person giving consent	Date	Signature

____

Relationship of person giving consent to participant

______________________	______________	______________
Name of person taking consent (if different from researcher)	Date	Signature

______________________	______________	______________
Researcher	Date	Signature

Copies: 1 for volunteer; 1 for researcher; 1 to be kept with hospital notes

An open-label, multi-center, randomized, 2-period cross-over study to assess the efficacy and safety of closed loop insulin delivery in comparison with sensor augmented insulin pump therapy with continuous glucose monitoring (CGM) over 4 months in children with type 1 diabetes aged 1 to 7 years in the home setting.

KidsAP02 Study

Statistical Analysis Plan

Randomized Crossover Trial

Version: 1.3

Version Date: 07/13/2020

Author: Nathan Cohen

Protocol Version: 1.2

Note: The table shells are included in a separate document.

Revision History

The following table outlines changes made to the Statistical Analysis Plan.

Version Number	Author	Approver	Effective Date	Study Stage
1.0	Nathan Cohen	Peter Calhoun	04/04/2019	Protocol development
1.1	Nathan Cohen	Peter Calhoun	06/19/2019	Enrollment
1.2	Nathan Cohen	Peter Calhoun	07/11/2019	Enrollment
1.3	Nathan Cohen	Peter Calhoun	07/13/2020	Follow-up

Version Number	Revision Description
1.1	Clarified when the end of the period occurs for safety analyses and that secondary analyses by time of day include the same subjects as the primary analysis
1.2	Clarified the scoring of the INSPIRE survey
1.3	Added in the questionnaires for the sleep sub-study. Changed the definition of daytime and nighttime to be consistent with the protocol.

Author: ________________________________

Senior Statistician: ________________________________

Jaeb Principal Investigator: ________________________________

Fuchs J, *et al. BMJ Open* 2021; 11:e042790. doi: 10.1136/bmjopen-2020-042790

1. Overview

This document outlines the statistical analyses to be performed for the KidsAP02 study. The approach to sample size and statistical analyses for this study are summarized below.

This is an open-label, multicenter, randomized, two period crossover study to assess the efficacy and safety of closed loop (CL) insulin delivery in comparison with sensor augmented/insulin pump (SAP) therapy with continuous glucose monitoring (CGM) over four months in children with type I diabetes aged 1-7 years. Approximately 72 subjects are expected to be randomized and enter the trial. All participants will receive both interventions, and the order of receiving them will be randomized based on a 1:1 ratio. Randomization will be preceded by a 2-4 week run-in period where subjects must demonstrate competency and compliance in using the study insulin pump and CGM device. After randomization, the subjects will enter the two four month study periods and will test one intervention per study period. The two periods will be separated by a 1-4 week washout period.

2. Statistical Hypotheses

- *Null Hypothesis:* There is no difference in the true mean time spent in the target range (3.9 to 10.0 mmol/L) over the four month period between the two treatment groups.
- *Alternative Hypothesis:* There is a nonzero difference in the true mean time spent in the target range over the four month period between the two treatment groups.

3. Sample Size

The study is projected to randomize 72 subjects. The sample size was calculated assuming 90% power, a treatment effect of 5% in the percentage of time in the target range, a standard deviation of 10.3% for an individual measurement, and a correlation of 0.3 between periods.

4. Outcome Measures

Primary Efficacy Endpoint:

Time spent in the target range (3.9 to 10.0 mmol/L) over the four month period

Other Key Endpoints:

1) Percent Time spent with glucose levels above 10.0 mmol/L
2) HbA1c
3) Mean of glucose levels
4) Percent Time spent with glucose levels below 3.9 mmol/L

Secondary Efficacy Endpoints:

CGM Metrics

Overall Glucose Control

1) Standard deviation of glucose levels
2) Coefficient of variation of glucose levels

Hyperglycemia

3) Percent Time spent with glucose levels above 16.7 mmol/L

Hypoglycemia

4) Percent Time spent with glucose levels below 3.5 mmol/L

5) Percent Time spent with glucose levels below 3.0 mmol/L
6) Area under the curve of glucose levels below 3.5 mmol/L
7) Area under the curve of glucose levels below 3.0 mmol/L

Insulin Delivery

8) Total insulin dose
9) Basal insulin dose
10) Bolus insulin dose

Clinical Metrics

11) BMI standard deviation score

Questionnaires

12) Strengths and Difficulties Questionnaire
    - Emotional Problems Subscale
    - Conduct Problems Subscale
    - Hyperactivity Subscale
    - Peer Problems Subscale
    - Prosocial Subscale
    - Total Difficulties Score
13) Hypoglycemia Fear Survey
    - Behavior Subscale
    - Worry Subscale
14) WHO-5
15) Epworth Sleepiness Scale
16) Pittsburgh Sleep Quality Index
    - Subjective Sleep Quality Subscale
    - Sleep Latency Subscale
    - Sleep Duration Subscale
    - Habitual Sleep Efficiency Subscale
    - Sleep Disturbances Subscale
    - Use of Sleeping Medication Subscale
    - Daytime Dysfunction Subscale
17) Children's Sleep Habit Questionnaire
    - Bedtime Resistance Subscale
    - Sleep Onset Delay Subscale
    - Sleep Duration Subscale
    - Sleep Anxiety Subscale
    - Night Waking Subscale
    - Parasomnias Subscale
    - Sleep Disordered Breathing Subscale
    - Daytime Sleepiness Subscale

4.1 Calculation of CGM Metrics

For the primary outcome and all secondary CGM metrics, a single value will be calculated for each subject for each period by pooling all CGM readings between the treatment initiation visit and up to 112 days post-initiation visit or the end of treatment visit, whichever comes first. All glucose sensor readings will be weighted equally in the pooled percentages regardless of how they distribute across weeks. Data will not be truncated due to protocol deviations.

Baseline CGM metrics will be calculated by pooling all readings up to the last 14 available days of CGM readings prior to randomization.

4.2 Calculation of BMI SD Scores

BMI standard deviation score will be calculated using the WHO growth chart.

5. Analysis Datasets and Sensitivity Analyses

5.1 Analysis Cohorts

- The primary analysis and all secondary analyses will be performed on a modified intention-to-treat basis with each day included in the treatment group assigned by randomization.
- A per-protocol analysis restricted to randomized participants with a minimum of 60% of available CGM readings during the control period and 60% CL system use during the CL period will be conducted for the primary outcome.
- Safety outcomes will be reported for all enrolled participants, regardless of whether the study was completed.

6. Analysis of the Primary Efficacy Endpoint

6.1 Included Subjects

Only subjects with at least 168 hours of CGM data in at least one period will be included. If a subject has more than 168 hours of data in period 1 and then drops out of the study without any data in period 2, then he or she will be included in the analysis.

6.2 Missing Data

Missing data will not be imputed for the primary analysis in this study.

6.3 Statistical Methods

Mean ± SD or summary statistics appropriate to the distribution will be reported for the primary outcome and each of the key secondary outcomes listed below over the four month period by treatment intervention. The treatment interventions will be compared using a linear mixed model adjusting for period as a fixed effect and site as a random effect. The analysis dataset will be three records per subject (one for baseline and one for each period). Inclusion of the pre-randomization baseline value as a third observation for each subject in the model gives a variance reduction analogous to adjusting for it as a covariate. Baseline is not modeled as a covariate in this analysis because there is no corresponding baseline for period 2, only pre-randomization. Note that adjusting for a post-randomization period 2 baseline can introduce a bias so that is not done here. The model will account for correlated data from the same subject. A 95% confidence interval will be reported for the difference between the interventions based on the linear mixed model.

Residual values will be examined for an approximate normal distribution. If values are highly skewed, then a ranked normal score transformation will be used instead. However, previous experience suggests that the primary outcome will follow an approximately normal distribution. A separate model will also be built with the inclusion of a period by treatment interaction to

assess for the presence of a carryover effect. We do not expect a carryover effect to be present. A two-sided p-value will be reported.

For the primary endpoint and other key endpoints listed in section 4, the familywise type I error rate (FWER) will be controlled at two-sided $\alpha = 0.05$. A gatekeeping strategy will be used, where the primary endpoint will be tested first, if passing the significance testing, other key endpoints will be tested in the order listed below using the fixed-sequence method at $\alpha = 0.05$:

- Time spent with sensor glucose levels between 3.9 to 10.0 mmol/l
- Time spent above target glucose (10.0 mmol/l)
- HbA1c
- Average of glucose levels
- Time spent below target glucose (3.9 mmol/l)

This process continues iteratively moving to the next variable down on the list until a non-significant result ($p \geq 0.05$) is observed, or all five variables have been tested. If a non-significant result is encountered, then formal statistical hypothesis testing is terminated and any variables below on the list are not formally tested and analysis of these variables becomes exploratory.

For example, in the hypothetical scenario depicted in the table below, the first three outcome variables both have a significant result so testing continues to the fourth variable (CGM mean glucose). The result is not significant for that fourth variable ($p = 0.33$) so testing stops. No formal hypothesis test is conducted for the fifth variable on the list in this example scenario.

HIERARCHICAL ORDER	OUTCOME VARIABLE	TREATMENT ARM P-VALUE	SIGNIFICANT?	ACTION
1st	CGM % 3.9-10.0 mmol/L (primary outcome)	0.001	Yes	Test next variable
2nd	CGM % above 10.0 mmol/L	0.02	Yes	Test next variable
3rd	HbA1c	0.007	Yes	Test next variable
4th	CGM mean glucose	0.33	No	Stop formal testing
5th	CGM % below 3.9 mmol/L	Not tested	Unknown	N/A

Regardless of the results of the hierarchical testing, summary statistics appropriate to the distribution will be tabulated by treatment arm for each hierarchical outcome. A 95% confidence interval for the treatment arm difference will also be calculated for all five hierarchical outcomes listed above. However, a confidence interval that excludes zero will not be considered a statistically significant result if an outcome variable higher on the hierarchical list failed to reach statistical significance.

7. Analysis of the Secondary Endpoints

7.1 Included Subjects

In the analyses involving HbA1c and BMI standard deviation score, all subjects with an available measurement within the analysis windows specified in section 7.3 will be included.

For secondary CGM metrics, inclusion criteria will be the same as the primary analysis.

For the secondary insulin outcomes, at least 168 hours of insulin data in at least one period will be required for inclusion. If a subject has more than 168 hours of insulin data in period 1 and then drops out of the study without any data in period 2, then he or she will be included in the analysis.

7.2 Missing Data

For the secondary CGM and insulin metrics, missing data will not be imputed in this study.

In the event that a BMI standard deviation score or HbA1c is unavailable at the end of a period but is available at a previous time point, it will be estimated using the method of direct likelihood to incorporate information from previous measurements to calculate the maximum likelihood estimate.

It is worth noting that all statistical methods for handling missing data rely on untestable assumptions and there is no one correct way to handle missing data. Our goal is to minimize the amount of missing data so that the results will not be sensitive to which statistical method is used.

7.3 Analysis Windows

Only HbA1c and body weight measurements obtained within ±7 days of the end of treatment visit dates during each period will be included in the analyses as the outcome. The baseline measurements must be within ±14 days of the recruitment visit.

7.4 Statistical Methods

7.4.1 Secondary CGM Outcomes

For all secondary CGM outcomes, summary statistics appropriate to the distribution will be tabulated by treatment group over the four month period. Analysis of all secondary CGM endpoints will parallel the primary analysis. A ranked normal score transformation will be applied to all highly skewed secondary outcomes.

7.4.2 Secondary Insulin Outcomes

For all secondary insulin outcomes, summary statistics appropriate to the distribution will be tabulated by treatment group over the four month period. Analysis of all secondary insulin endpoints will parallel the primary analysis. A ranked normal score transformation will be applied to all highly skewed secondary outcomes.

7.4.3 Secondary HbA1c Outcomes

For HbA1c, a longitudinal model adjusting for period as a fixed effect will be constructed to compare treatment arms. The model will include three time points: (1) baseline, (2) period 1 outcome, and (3) period 2 outcome.

7.4.4 Secondary Clinical Outcomes

Fuchs J, *et al. BMJ Open* 2021; 11:e042790. doi: 10.1136/bmjopen-2020-042790

For BMI standard deviation score, a longitudinal model adjusting for period as a fixed effect will be constructed to compare treatment arms. The model will include three time points: (1) baseline, (2) period 1 outcome, and (3) period 2 outcome.

7.5 Secondary Analyses by Time of Day

Summary statistics for the following outcome metrics will also be tabulated separately for daytime (defined as 8am to less than 12am) and nighttime (defined as 12am to less than 8am) over the four month period:

- Percent time with glucose levels spent in the target range (3.9 to 10.0 mmol/L)
- Mean of glucose levels
- Standard deviation of glucose levels
- Percent time with glucose levels below 3.9 mmol/L
- Total insulin dose

For each of these outcome metrics, the same model described above for the primary and secondary analyses will be fit with the inclusion of a treatment by time of day interaction. The same subjects will be included as in the primary analysis. The p-value for the interaction term will be reported. These analyses will be conducted to determine whether a similar trend to the overall treatment effect is seen in the different times of day.

The study is not expected to have sufficient statistical power for definitive conclusions in the secondary analyses by time of day, and statistical power will be low to formally assess for the presence of a treatment by time of day interaction. Interpretation of the analyses by time of day will depend on whether the overall analysis demonstrates a significant treatment effect. In the absence of any significant treatment effects in the overall analyses, assessment of secondary analyses by time of day will be considered exploratory and used to suggest hypotheses for further investigation in future studies.

7.6 Questionnaire Analyses

For each questionnaire (and their corresponding subscales), total scores will be calculated and reported at each time point. They will also be compared between treatment arms using the same model described above for the primary outcome. The distribution of responses for each individual question at baseline and for each treatment arm will also be reported in separate tables.

For the INSPIRE Survey, a mean score will be calculated instead of a total score. The mean score will be calculated after excluding all questions where the participants give a response of "NA".

For the Pittsburgh Sleep Quality Index (and all of its subscales), the scoring instructions will be used to calculate the total scores overall and by subscale. For this questionnaire, scores will be tabulated for each parent who completes the survey.

For the INSPIRE Survey and the CL Experience Survey, a treatment arm comparison will not be done, because the surveys are only completed at the end of the CL arm. For these questionnaires, only summary statistics for the scores and the distribution of responses for each question will be

Fuchs J, *et al. BMJ Open* 2021; 11:e042790. doi: 10.1136/bmjopen-2020-042790

reported. Additionally, treatment arm comparisons will not be done for the Pittsburgh Sleep Quality Index or the Children's Sleep Habit Questionnaire, because they are only completed at visit 8.

For the Strengths and Difficulties Questionnaire, no overall total score will be calculated that involves every question. Instead, only a Total Difficulties Score is computed from the questions comprising the Emotional Problems, Conduct Problems, Hyperactivity, and Peer Problems subscales.

The electronic data capture system for this study will not allow the subject to submit any questionnaires until all items are completed. Analysis will be limited to subjects who submit a questionnaire (no imputation).

7.7 Ancillary Analyses

Cost utility analyses and human factors analyses will be described in a separate document.

8. Safety Analyses

All safety outcomes will be tabulated by participant for all events from enrollment to the final study visit.

8.1 Definitions

Reportable adverse events for this protocol include any untoward medical occurrence, unintended disease or injury, or untoward clinical signs (including abnormal laboratory findings) in a subject who has received an investigational device, whether or not related to the investigational medical device. These include severe hypoglycemia (SH) and diabetic ketoacidosis (DKA).

Hypoglycemic events will be considered severe if the event requires assistance of another person due to altered consciousness to actively administer carbohydrate, glucagon, or other resuscitative actions. This means that the subject is impaired cognitively to the point that he/she is unable to treat him- or herself, is unable to verbalize his or her needs, is incoherent, disoriented, and/or combative, or experiences seizure or coma. If plasma glucose measurements are not available during such an event, neurological recovery attributable to the restoration of plasma glucose to normal is considered sufficient evidence that the event was induced by a low plasma glucose concentration.

Definite DKA is defined as having all of the following:

- Hyperglycemia (blood glucose >200 mg/dL or >11 mmol/L)
- with either low pH (<7.3) or low serum bicarbonate (<15 mmol/L)
- and ketonemia or ketonuria

8.2 Adverse Events Summary

All episodes of SH and of DKA along with any other reportable adverse events will be listed by treatment group.

Separate listings will be provided for pre-randomization and post-randomization adverse events.

8.3 Comparison of Safety Outcomes between Treatment Groups

Fuchs J, *et al. BMJ Open* 2021; 11:e042790. doi: 10.1136/bmjopen-2020-042790

The following safety analyses will be performed if enough events occur for formal statistical analyses.

For each of the following safety outcomes, mean ± SD or summary statistics appropriate to the distribution will be tabulated by treatment group:

- Number of subjects with any DKA events
- Number of episodes of DKA events per subject and incidence rate per 100 person years
- Number of subjects with any SH events
- Number of episodes of SH events per subject and incidence rate per 100 person years
- Number of adverse events per subject
- Number of serious adverse events per subject

All of the above safety outcomes will be tabulated for all subjects (including dropouts and withdrawals), regardless of whether CGM data are available or whether the closed loop system was operational (if the event occurred during the CL period). Any adverse events that occurred before the treatment initiation visit in period 1 or during the washout period will not be included in the rate calculations or treatment group comparisons listed above. In all safety analyses, each period will inclusively consist of all days in between the treatment initiation visit and the end of treatment visit. If the subject drops out of the study in the middle of a period and the end of treatment visit for that particular period does not occur, then the dropout date will be used as the last day of the period.

The number of person-years for the incidence rate calculations in each period will be inclusively defined as the number of person-years in between the treatment initiation visit date and the end of treatment visit date. If the subject drops out of the study in the middle of a period and the end of treatment visit for that particular period does not occur, then the dropout date will be used as the last day of the period.

For each of DKA and SH (if enough events), the event rates will be compared using a repeated measures Poisson regression model adjusting for period and whether the subject has ever had a prior event. Binary variables will also be compared using a repeated measures logistic regression model adjusting for period and whether the subject has ever had a prior event.

9. Adherence and Retention Analyses

9.1 Utility Analysis

The amount of CGM use will be tabulated for each treatment arm, in addition to the amount of closed loop system use in the CL arm. Summary statistics appropriate to the distribution and range will be reported for the percentage of time using the CGM over the four month period (as defined above) for each treatment group. The same will be done for the percentage of time using the closed loop system in the CL arm. Tabulations of summary statistics will also be performed for the percentage of time spent using the closed loop system while using the CGM in the CL arm.

The percentage of time spent using the CGM will be calculated by dividing the total number of CGM readings by the expected number of readings during the four-month period. The

Fuchs J, *et al. BMJ Open* 2021; 11:e042790. doi: 10.1136/bmjopen-2020-042790

percentage of time using the closed loop system in the CL arm will be calculated by dividing the total amount of time that temporary basal infusion lasts no more than 30 minutes by the maximum possible amount of time that the system could have been used. The percentage of time using the closed loop system while using the CGM (in the CL arm) will then be computed by dividing the time that the closed loop system was operational by the amount of time that the CGM was available.

If a subject drops out of the study in the middle of a period, then the subject will be counted as not using the CGM or the closed loop system at all during the remainder of the study. Thus, these time points will be counted as zero use in the calculation of CGM use and closed loop system use.

9.2 Protocol Adherence and Retention

The following tabulations and analyses will be performed to assess protocol adherence for the study:

- Number of protocol and procedural deviations per subject along with the number and percentage of subjects with each number of deviations
- Number of protocol and procedural deviations by severity with brief descriptions listed
- Flow chart accounting for all subjects at all visits post randomization to assess visit completion rates
- A flow chart accounting for the number of subjects enrolled, the number of dropouts pre- and post-randomization, and the number of subjects eligible to be included in the primary analysis
- Number of and reasons for unscheduled visits

10. Baseline Descriptive Statistics

Baseline demographic and clinical characteristics of the cohort of all randomized subjects will be summarized in a table. Descriptive statistics will be tabulated overall and by randomization group. For continuous variables, summary statistics appropriate to the distribution will be given. For discrete variables, number and percentage will be reported for each category. The following baseline CGM metrics will be included in the table:

- % Time in Range (3.9-10.0 mmol/L)
- Mean of sensor glucose levels
- Standard deviation of glucose levels
- Coefficient of variation of glucose levels
- % Time >10.0 and >16.7 mmol/L
- % Time <3.9, <3.5, and <3.0 mmol/L
- Area under the curve of glucose levels below 3.5 mmol/L and below 3.0mmol/L

11. Planned Interim Analyses

No formal interim analyses or stopping guidelines are planned for this study.

The DSMB will review data collected for the study every six months. The data to be reviewed will include information regarding all of the following:

- Status of randomized participants
- Recruitment rates by month and by site

Fuchs J, *et al. BMJ Open* 2021; 11:e042790. doi: 10.1136/bmjopen-2020-042790

- Baseline demographic and clinical characteristics
- Dropped participants and reasons for discontinuing
- Reportable adverse events

12. Subgroup Analyses

No subgroup analyses are planned for this study.

13. Multiple Comparisons/Multiplicity

13.1 Primary analysis and other key secondary outcomes

For the primary endpoint and other key endpoints listed in section 4, the familywise type I error rate (FWER) will be controlled at two-sided $\alpha = 0.05$. A gatekeeping strategy will be used, where the primary endpoint will be tested first, if passing the significance testing, other key endpoints will be tested in the order listed below using the fixed-sequence method at $\alpha = 0.05$:

- Time spent with sensor glucose levels between 3.9 to 10.0 mmol/l (70 to 180 mg/dl)
- Time spent above target glucose (10.0 mmol/l) (180 mg/dl)
- HbA1c
- Average of glucose levels
- Time spent below target glucose (3.9 mmol/l) (70 mg/dl)

Additional details are provided in section 6.3.

13.2 Other Secondary Analyses

For the other secondary endpoints listed in section 4, Benjamini-Hochberg false discovery rate (FDR) adjusted p-values will be calculated within each subcategory below:

CGM derived indices:

- Standard deviation, and coefficient of variation of glucose levels
- Time with glucose levels <3.5 mmol/L (63 mg/dL) and <3.0 mmol/l (54 mg/dl)
- Time with glucose levels in significant hyperglycaemia (glucose levels > 16.7 mmol/l) (300 mg/dl)
- AUC of glucose below 3.5 mmol/l (63 mg/dl) and below 3.0mmol/l (54mg/dl)

Insulin and Other Endpoints:

- BMI standard deviation score
- Total, basal, and bolus insulin dose

Questionnaires:

- Strengths and Difficulties Questionnaire
  - Emotional Problems Subscale
  - Conduct Problems Subscale
  - Hyperactivity Subscale
  - Peer Problems Subscale
  - Prosocial Subscale
  - Total Difficulties Score
- Hypoglycemia Fear Survey
  - Behavior Subscale

  - Worry Subscale
- WHO-5
- Epworth Sleepiness Scale

Analyses by Time of Day:

- Percent time with glucose levels spent in the target range (3.9 to 10.0 mmol/L)
- Mean of glucose levels
- Standard deviation of glucose levels
- Percent time with glucose levels below 3.9 mmol/L
- Total insulin dose

14. Exploratory Analyses

No exploratory analyses will be performed for this study.

Fuchs J, *et al. BMJ Open* 2021; 11:e042790. doi: 10.1136/bmjopen-2020-042790

Clinical Study Protocol

**Study Title:**	**An open-label, multi-centre, multi-national, randomised, 2-period crossover study to assess the efficacy, safety and utility of closed loop insulin delivery in comparison with sensor augmented pump therapy over 4 months in children with type 1 diabetes aged 1 to 7 years in the home setting.**
**Short Title:**	**The artificial pancreas in very young children with T1D (KidsAP02)**
**Protocol Version:**	2.0 10 March 2020

**Chief Investigator**	Dr Roman Hovorka<br>Wellcome Trust-MRC Institute of Metabolic Science<br>University of Cambridge<br>Box 289, Level 4<br>Addenbrooke's Hospital<br>Hills Road, Cambridge<br>CB2 0QQ<br>UK<br>Phone: +44 1223 762 862<br>Fax: +44 1223 336 996<br>E-mail: rh347@cam.ac.uk

This protocol has been written in accordance with current ISO 14155:2011 standard

Fuchs J, *et al. BMJ Open* 2021; 11:e042790. doi: 10.1136/bmjopen-2020-042790

**Principal Clinical Investigators**	Dr Ajay Thankamony<br>Children's Services<br>Box 267<br>Addenbrooke's Hospital, Hills Road<br>Cambridge, CB2 0QQ<br>United Kingdom<br>Phone: 01223 274311<br>Email: ajay.thankamony@addenbrookes.nhs.uk<br><br>Dr Carine de Beaufort<br>Clinique Pédiatrique de Luxembourg<br>Centre Hospitalier de Luxembourg<br>4 rue Ernest Barblé<br>L-1210 Luxembourg<br>Luxembourg<br>Phone: +352 44113133<br>Fax: +352 44116466<br>E-mail: bcschim@pt.lu<br><br>Dr Fiona Campbell<br>St James's University Hospital<br>Beckett Street<br>Leeds, LS9 7TF<br>United Kingdom<br>Phone: +44 113 218 5908/9<br>E-mail: Fiona.Campbell26@nhs.net<br><br>Dr Elke Fröhlich-Reiterer<br>Medical University of Graz<br>Department of Pediatrics and Adolescent Medicine<br>Auenbruggerplatz 34/2<br>A-8036 Graz<br>Austria<br>Phone: +43 316 385 80562<br>Fax: +43 316 385 13896<br>E-mail: elke.froehlich-reiterer@medunigraz.at<br><br>Dr Sabine Hofer<br>Medical University of Innsbruck<br>Department of Pediatrics I<br>Anichstrasse 35<br>A-6020 Innsbruck<br>Austria<br>Phone: +43 512 504 23600<br>Fax: +43 512 504 25450<br>E-mail: Sabine.E.Hofer@i-med.ac.at

Fuchs J, *et al. BMJ Open* 2021; 11:e042790. doi: 10.1136/bmjopen-2020-042790

	Dr Thomas Kapellen<br>University of Leipzig<br>Division for Paediatric Diabetology<br>Liebigstr. 20<br>D-04103 Leipzig<br>Germany<br>Phone: +49 341 97 109<br>Fax: +49 341 97 12 329<br>E-mail: ThomasMichael.Kapellen@medizin.uni-leipzig.de<br><br>Dr Birgit Rami-Merhar<br>Medical University of Vienna<br>Deptartment of Pediatrics<br>Waehringer Gürtel 18-20<br>A-1090 Vienna<br>Austria<br>Phone: +43-1-40400-32320<br>Fax: +43-1-40400-59850<br>E-mail: Birgit.Rami@meduniwien.ac.at
**Clinical Investigators**	Dr Heike Bartelt<br>University of Leipzig<br>University of Leipzig<br>Division for Paediatric Diabetology<br>Liebigstr. 20<br>D-04103 Leipzig<br>Germany<br>Phone: +49 341 97 109<br>Fax: +49 341 97 12 329<br>E-mail: Heike.Bartelt@medizin.uni-leipzig.de<br><br>Dr Charlotte Boughton<br>Wellcome Trust-MRC Institute of Metabolic Science<br>University of Cambridge<br>Box 289, Level 4<br>Addenbrooke's Hospital, Hills Road<br>Cambridge, CB2 0QQ<br>United Kingdom<br>Phone: +44 1223 769 066<br>Fax: +44 1223 336 996<br>Email: cb2000@medschl.cam.ac.uk<br><br>Dr Elisabeth Binder<br>Medical University of Innsbruck<br>Department of Pediatrics I

Fuchs J, *et al. BMJ Open* 2021; 11:e042790. doi: 10.1136/bmjopen-2020-042790

	Anichstrasse 35<br>A-6020 Innsbruck<br>Austria<br>Phone: +43 512 504 23600<br>Fax: +43 512 504 25450<br>E-mail: Elisabeth.Binder@i-med.ac.at<br><br>Dr Maria Fritsch<br>Medical University of Vienna<br>Deptartment of Pediatrics<br>Waehringer Gürtel 18-20<br>A-1090 Vienna<br>Austria<br>Phone: +43-1-40400-32320<br>Fax: +43-1-40400-59850<br>E-mail: maria.fritsch@meduniwien.ac.at<br><br>Dr Lukas Hackl<br>Medical University of Innsbruck<br>Department of Pediatrics I<br>Anichstrasse 35<br>A-6020 Innsbruck<br>Austria<br>Phone: +43 512 504 23600<br>Fax: +43 512 504 25450<br>E-mail: Lukas.Hackl@i-med.ac.at<br><br>Dr Hildegard Jasser-Nitsche<br>Medical University of Graz<br>Department of Paediatrics and Adolescent Medicine<br>Division of Endocrinology and Diabetes<br>Auenbruggerplatz 34<br>A - 8036 Graz, Austria<br>Tel. +43 316 385 82616<br>Fax. +43 316 385 13896<br>E-mail: Hildegard.nitsche@medunigraz.at<br><br>Dr Katrin Nagl<br>Medical University of Vienna<br>Deptartment of Pediatrics<br>Waehringer Gürtel 18-20<br>A-1090 Vienna<br>Austria<br>Phone: +43-1-40400-32320<br>Fax: +43-1-40400-59850<br>E-mail: katrin.nagl@meduniwien.ac.at

Fuchs J, et al. BMJ Open 2021; 11:e042790. doi: 10.1136/bmjopen-2020-042790

	Dr Julia Mader<br>Medical University of Graz,<br>Dept. of Internal Medicine<br>Division of Endocrinology and Diabetology<br>Auenbruggerplatz 15<br>A- 8036 Graz<br>Austria<br>Phone: +43 316 385 80254<br>Fax: +43 316 385 13428<br>E-mail: julia.mader@medunigraz.at<br><br>Dr Julia Fuchs<br>Wellcome Trust-MRC Institute of Metabolic Science<br>Box 289, Level 4<br>Addenbrooke's Hospital<br>Hills Road<br>Cambridge CB2 0QQ<br>UK<br>Tel: +44 1223 769 069<br>Fax: +44 1223 336 996<br>Email: jf674@medschl.cam.ac.uk<br><br>Dr Ulrike Schierloh<br>Clinique Pédiatrique de Luxembourg<br>Centre Hospitalier de Luxembourg<br>4 rue Ernest Barblé<br>L-1210 Luxembourg<br>Luxembourg<br>Phone: +352 44113133<br>Fax: +352 44116466<br>E-mail: Schierloh.Ulrike@chl.lu<br><br>Dr Elisabeth Steichen<br>Medical University of Innsbruck<br>Department of Pediatrics I<br>Anichstrasse 35<br>A-6020 Innsbruck<br>Austria<br>Phone: +43 512 504 23600<br>Fax: +43 512 504 25450<br>E-mail: elisabeth.steichen@i-med.ac.at<br><br>Dr Elisabeth Suppan<br>Medical University of Graz<br>Department of Pediatrics and Adolescent Medicine

	Auenbruggerplatz 34<br>A-8036 Graz<br>Austria<br>Phone: +43 316 385 80254<br>Fax: +43 316 385 13428<br>E-mail: elisabeth.suppan@klinikum-graz.at<br><br>Dr Martin Tauschmann<br>Medical University of Vienna<br>Department of Pediatrics<br>Waehringer Gürtel 18-20<br>A-1090 Vienna<br>Austria<br>Phone: +43-1-40400-32320<br>Fax: +43-1-40400-59850<br>E-mail: martin.tauschmann@meduniwien.ac.at<br><br>Dr Rachel Williams<br>Cambridge University Hospitals NHS Foundation Trust<br>The Weston Centre for Paediatric Diabetes and Endocrinology<br>Box 280<br>Addenbrooke's Hospital, Hills Road Cambridge, CB2 0QQ<br>United Kingdom<br>Phone: +44 1223 245151 ext 4311<br>E-mail: rmw33@medschl.cam.ac.uk<br><br>Dr James Yong<br>St James's University Hospital<br>Beckett Street<br>Leeds, LS9 7TF<br>United Kingdom<br>Phone: +44 113 2066897<br>E-mail: james.yong@nhs.net
**Other Investigators**	Dr Malgorzata E Wilinska<br>Wellcome Trust-MRC Institute of Metabolic Science<br>University of Cambridge<br>Box 289, Level 4<br>Addenbrooke's Hospital<br>Hills Road, Cambridge<br>CB2 0QQ<br>United Kingdom<br>Phone: +44 1223 769 065<br>Fax: +44 1223 336 996<br>E-mail: mew37@cam.ac.uk

Fuchs J, *et al. BMJ Open* 2021; 11:e042790. doi: 10.1136/bmjopen-2020-042790

**Medical Sociologist**	Professor Julia Lawton<br>Centre for Population Health Sciences<br>The University of Edinburgh<br>Teviot Place<br>Edinburgh, EH8 9AG<br>UK<br>Tel: +44 (0)131 650 6197<br>Fax: +44 (0)131 650 6902<br>E-mail: J.Lawton@ed.ac.uk
**Health Economist**	Stéphane Roze<br>Expert Health-Economist<br>HEVA HEOR Sarl<br>186 avenue Thiers<br>69006 Lyon<br>France<br>Tel: +33 (0)4 72 14 52 43<br>E-mail: SROZE@heva-heor.com
**Human Factor Assessment**<br><br><br><br><br><br><br><br><br>**Sleep Quality Assessment**	Professor Korey K. Hood<br>Stanford University School of Medicine<br>291 Campus Drive<br>Li Ka Shing Building<br>Stanford, CA 94305-5101<br>USA<br>Tel:+1 (650) 497-6899<br>E-mail: kkhood@stanford.edu<br><br>Professor Alice M Gregory<br>Department of Psychology<br>Goldsmiths, University of London<br>New Cross<br>London, SE14 6NW<br>UK<br>Tel: +44 (0)2079 197 959<br>E-mail: A.Gregory@gold.ac.uk
**Study Co-ordinator**	Ms Nicole Barber<br>Wellcome Trust-MRC Institute of Metabolic Science<br>Box 289, Level 4<br>Addenbrooke's Hospital<br>Hills Road, Cambridge CB2 0QQ<br>United Kingdom<br>Phone: +44 1223 769 068

Fuchs J, *et al. BMJ Open* 2021; 11:e042790. doi: 10.1136/bmjopen-2020-042790

<table>
<tr><td></td><td colspan="2">Fax +44 1223 330 598
E-mail: nlb39@medschl.cam.ac.uk</td></tr>
<tr><td>Study Statistician</td><td colspan="2">Dr Craig Kollman
Jaeb Centre for Health Research
15310 Amberly Drive, Suite 350
Tampa, FL 33647
USA
Phone: +1 (813) 975-8690
Fax: +1 (813) 903-8227
E-mail: ckollman@Jaeb.org</td></tr>
<tr><td>Study Data Manager</td><td colspan="2">Mr Chris McCarthy
Jaeb Centre for Health Research
15310 Amberly Drive, Suite 350
Tampa, FL 33647
USA
Tel: +1 (813) 975-8690
Fax: +1 (813) 903-8227
E-mail: cmccarthy@Jaeb.org</td></tr>
<tr><td rowspan="2">Study Sponsor</td><td colspan="2">Cambridge University Hospitals NHS Foundation Trust, jointly with University of Cambridge</td></tr>
<tr><td>Ms Carolyn Read
Research Governance Office
University of Cambridge
School of Clinical Medicine
Box 111, Cambridge Biomedical Campus
Cambridge, CB2 0SP
UK
Tel: +44 (0)1223 769 291
E-mail:
researchgovernance@medschl.cam.ac.uk</td><td>Stephen Kelleher
Cambridge University Hospitals NHS Foundation Trust
Box 277, Addenbrooke's Hospital
Hills Road, Cambridge, CB2 0QQ, UK
Phone: +44 1223 217 418
Fax: +44 1223 348 494
E-mail:
enquiries@addenbrookes.nhs.uk</td></tr>
<tr><td>Collaborating Organisation</td><td colspan="2">Glooko (Diasend)
Nellickevägen 20
412 63 Göteborg
Sweden</td></tr>
<tr><td>Funder</td><td colspan="2">European Commission within the Horizon2020 Framework Programme</td></tr>
</table>

Fuchs J, *et al. BMJ Open* 2021; 11:e042790. doi: 10.1136/bmjopen-2020-042790

PROTOCOL SIGNATURE PAGE

The signature below documents the approval of the protocol entitled **"An open-label, multi-centre, multi-national randomised, 2-period cross-over study to assess the efficacy, safety and utility of closed loop insulin delivery in comparison with sensor augmented pump therapy over 4 months in children with type 1 diabetes aged 1 to 7 years in the home setting."** Version _. _ dated _ _ / _ _ /_ _ _ _ and provides the necessary assurances that this study will be conducted according to all stipulations of the protocol, the principles of GCP and the appropriate reporting requirements.

Signature ……………………………………. Date……………………….

Dr Roman Hovorka, Chief Investigator

Fuchs J, et al. BMJ Open 2021; 11:e042790. doi: 10.1136/bmjopen-2020-042790

SITE SIGNATURE PAGE

I have read the attached protocol entitled " **An open-label, multi-centre, multi-national randomised, 2-period cross-over study to assess the efficacy, safety and utility of closed loop insulin delivery in comparison with sensor augmented pump therapy over 4 months in children with type 1 diabetes aged 1 to 7 years in the home setting"** Version _._ dated _ _/_ _ _/_ _, and agree to abide by all provisions set forth therein.

I agree to comply with the conditions and principles of Good Clinical Practice as outlined in the European Clinical Trials Directives 2001/20/EC and the GCP Directive 2005/28/EC.

I agree to ensure that the confidential information contained in this document will not be used for any other purpose other than the evaluation or conduct of the clinical investigation without the prior written consent of the Sponsor.

Signature ……………………………………. Date………………………
Name …………………………………….
Site, Country………………………….

Principal Clinical Investigator

Fuchs J, *et al. BMJ Open* 2021; 11:e042790. doi: 10.1136/bmjopen-2020-042790

Table of Contents

1 LIST OF ABBREVIATIONS AND RELEVANT DEFINITIONS 13
2 STUDY SYNOPSIS 15
3 SUMMARY 22
4 BACKGROUND 23
4.1 INTRODUCTION 23
4.2 CLOSED LOOP INSULIN DELIVERY 24
4.3 CLOSED LOOP RESEARCH IN CAMBRIDGE 25
Closed Loop Prototypes 25
Preliminary Data with the Cambridge Control Algorithm 26
4.4 KIDSAP CONSORTIUM 32
4.5 FLORENCEX CLOSED LOOP SYSTEM TO BE USED IN THE PRESENT STUDY 33
4.6 RATIONALE FOR THE PRESENT STUDY 33
5 OBJECTIVES 34
4.3.1 5.1 EFFICACY 34
4.3.2 5.2 SAFETY 34
5.3 UTILITY 34
5.4 HUMAN FACTORS 35
5.5 HEALTH ECONOMICS 35
6 STUDY DESIGN 35
7 STUDY SUBJECTS 37
7.1 STUDY POPULATION 37
Inclusion criteria 37
Exclusion criteria 37
7.2 RECRUITMENT 38
7.1.1 7.3 RANDOMISATION 39
8 *7.1.2* **METHODS UNDER INVESTIGATION 39**
8.1 NAME AND DESCRIPTION OF THE METHOD OF INVESTIGATION 39
8.2 INTENDED PURPOSE 39
8.3 METHOD OF ADMINISTRATION 39
8.4 REQUIRED TRAINING 40
8.5 PRECAUTIONS 40
8.6 ACCOUNTABILITY OF THE METHOD UNDER INVESTIGATION 40
9 STUDY SCHEDULE 40
9.1 OVERVIEW 40
9.2 RECRUITMENT VISIT (VISIT 1) 44
9.3 STUDY CGM TRAINING (VISIT 2) 44
9.4 STUDY PUMP TRAINING (VISIT 3) 45
9.5 RUN-IN 46
9.6 END OF RUN-IN, COMPLIANCE ASSESSMENT (VISIT 4) 46
9.8.1 9.7 RANDOMISATION 46
9.8.2 9.8 INITIATION OF STUDY ARM VISITS (VISIT 5, VISIT 7) 47
9.8.3 *Closed Loop Insulin Delivery 47*
Sensor augmented pump therapy 47
Remote Monitoring 47
9.9 TELEPHONE/MAIL CONTACTS AFTER STARTING STUDY TREATMENT (CONTACTS 2-11) 48
9.10 END OF 1ST STUDY ARM (VISIT 6) 48
9.11 WASHOUT 48
9.12 END OF 2ND STUDY ARM (VISIT 8) 49
9.13 PARTICIPANT WITHDRAWAL CRITERIA 49
9.14 STUDY STOPPING CRITERIA 50
9.15 CO-ENROLMENT GUIDELINES 50
9.16 SUPPORT TELEPHONE LINE 50
9.17 SUBJECT REIMBURSEMENT 51
10 ENDPOINTS 51
10.1 PRIMARY ENDPOINTS 51
10.2 OTHER KEY ENDPOINTS 51
11.1.1 10.3 SECONDARY ENDPOINTS 51
11.1.2 10.4 SAFETY EVALUATION 52
11.1.3 10.5 UTILITY EVALUATION 52
11.1.4 10.6 HUMAN FACTORS 52
11.1.5 10.7 HEALTH ECONOMICS 52
11 ASSESSMENT AND REPORTING OF ADVERSE EVENTS 52
11.1 DEFINITIONS 52
Reportable Adverse Events 53
Adverse Events 53
Adverse Device Effect 53
Serious Adverse Event 53
Serious Adverse Device Effect 54

Fuchs J, *et al. BMJ Open* 2021; 11:e042790. doi: 10.1136/bmjopen-2020-042790

Unanticipated Serious Adverse Device Effect *54*
Device Deficiencies *55*
Adverse Event Intensity *55*
Adverse Event Causality *55*
11.2 RECORDING AND REPORTING OF ADVERSE EVENTS, SERIOUS ADVERSE EVENTS AND DEVICE DEFICIENCIES 56
Monitoring Period of Adverse Events *56*
Recording and Reporting of Adverse Events *57*
11.1.6 *Severe Hypoglycaemia* *57*
11.1.7 *Diabetic Ketoacidosis* *57*
11.1.8 *Reporting of Serious Adverse Events and Serious Adverse Device Effects* *58*
11.1.9 *Recording and Reporting of Device Deficiencies* *59*
Healthcare Arrangements and Compensation for Adverse Events *60*
11.2.1 *Country specific requirements* *60*
11.2.2 11.3 ANTICIPATED ADVERSE EVENTS, RISKS AND BENEFITS 62
11.2.3
11.2.4 *Risks and Anticipated Adverse Events* *62*
11.2.5 *Hypoglycaemia and Hyperglycaemia* *62*
11.2.6 *Blood Sampling for HbA1c and Blood Glucose Measurements* *62*
11.2.7 *Insulin Pump Therapy* *62*
11.2.8 *Continuous Glucose Monitoring* *63*
11.3.1 *Questionnaires and Interviews* *63*
11.3.2 *Risk Analysis and Residual Risk Associated with the Investigational Device* *63*
11.3.3
11.3.4 11.4 BENEFITS 64
11.3.5 11.5 DATA SAFETY MONITORING BOARD (DSMB) 65
11.3.6 **12 METHODS AND ASSESSMENTS 65**
11.3.7 12.1 PROCEDURES 65
Height and Weight *65*
Blood Glucose Meter Data *65*
12.1.1 *Insulin Pump and CGM Data* *65*
12.1.2 12.2 LABORATORY METHODS 65
12.1.3 *HbA1c* *65*
Total Blood Loss *66*
12.2.1 12.3 QUESTIONNAIRES 66
12.2.2 12.4 QUALITATIVE INTERVIEW 67
12.5 HEALTH ECONOMICS 68
12.5.1 *Simulation cohort and treatment effects* *69*
12.5.2 *Costs and utilities* *69*
13 STUDY MATERIALS AND PRODUCTS 69
13.1 INSULIN 69
13.2 INSULIN PUMP 69
13.3 CONTINUOUS SUBCUTANEOUS GLUCOSE MONITOR 69
13.4 SMARTPHONE 70
13.5 BLOOD GLUCOSE METER 70
13.6 COMPUTER-BASED ALGORITHM 70
14 DATA ANALYSIS 70
14.1 PRIMARY ENDPOINT ANALYSIS 70
14.2 OTHER KEY ENDPOINTS 71
14.3 SECONDARY EFFICACY ANALYSES 72
14.4 SAFETY ANALYSES 73
14.5 UTILITY EVALUATION 73
14.6 QUESTIONNAIRES 74
14.7 INTERVIEWS 74
14.8 HEALTH ECONOMICS EVALUATION 75
14.9 INTERIM ANALYSIS 75
14.10 SAMPLE SIZE AND POWER CALCULATIONS 75
14.11 DEVIATIONS FROM THE STATISTICAL PLAN 76
15 CASE REPORT FORMS 76
16 DATA MANAGEMENT 76
17 ETHICS 77
17.1 INDEPENDENT RESEARCH ETHICS COMMITTEES (REC) 77
17.2 INFORMED CONSENT OF STUDY SUBJECTS 77
18 AMENDMENTS TO THE PROTOCOL 78
19 DEVIATIONS FROM PROTOCOL 78
20 STUDY MANAGEMENT 79
20.1 DATA AND SAFETY MONITORING BOARD (DSMB) 79
20.2 TRIAL STEERING COMMITTEE (TSC) 79
20.3 STUDY MONITORING 79
21 RESPONSIBILITIES 80
21.1 CHIEF INVESTIGATOR 80
21.2 PRINCIPAL CLINICAL INVESTIGATOR 80
21.3 STUDY COORDINATOR 80

22 REPORTS AND PUBLICATIONS........80
23 TIMETABLE........81
24 RETENTION OF STUDY DOCUMENTATION........81
25 INDEMNITY STATEMENTS........81
26 REFERENCES........82
27 DOCUMENT AMENDMENT HISTORY........88

1 List of Abbreviations and Relevant Definitions

ADE	Adverse Device Effect
AE	Adverse Event
AP	Artificial Pancreas
AR	Adverse Reaction
ASADE	Anticipated Serious Adverse Device Effect
AUC	Area under the Curve
CE	Conformité Européenne (CE-mark)
CGM	Continuous Glucose Monitoring
CI	Chief Investigator or Confidence Interval
CL	Closed-loop
CRF	Case Report Form
CSHQ	Children's Sleep Habit Questionnaire
CSII	Continuous subcutaneous insulin infusion
DKA	Diabetic Ketoacidosis
DSMB	Data Safety and Monitoring Board
EC	European Commission
eCRF	Electronic Case Report Form
EU	European Union
FDA	US Food and Drug Administration
GCP	Good Clinical Practice
H2020	Horizon 2020: The EU Framework Programme for Research and Innovation
HbA1c	Glycated Haemoglobin A1c
HIPAA	Health Insurance Portability and Accountability Act
IFCC	International Federation of Clinical Chemistry and Laboratory Medicine
ISPAD	International Society for Pediatric and Adolescent Diabetes
JDRF	Juvenile Diabetes Research Foundation
MHRA	Medicine and Healthcare Products Regulatory Agency
NGSP	National Glycohemoglobin Standardization Program
NHS	National Health Service
PIC	Participant Identification Centre

**PLGM**	**Predictive Low Glucose Management**
**PSQI**	**Pittsburgh Sleep Quality Index**
**R & D**	**Research and Development**
**RCT**	**Randomised Controlled Trial**
**REC**	**Research Ethics Committee**
**SADE**	**Serious Adverse Device Effect**
**SAE**	**Serious Adverse Event**
**SAP**	**Sensor Augmented Pump Therapy**
**SD**	**Standard Deviation**
**SMBG**	**Self-Monitoring of Blood Glucose**
**T1D**	**Type 1 Diabetes Mellitus**
**USADE**	**Unanticipated Serious Adverse Device Effect**
**WHO**	**World Health Organization**

Fuchs J, *et al. BMJ Open* 2021; 11:e042790. doi: 10.1136/bmjopen-2020-042790

2 Study Synopsis

**Title of clinical trial**	An open-label, multi-centre, multi-national, randomised, 2-period cross-over study to assess the efficacy, safety and utility of closed loop insulin delivery in comparison with sensor augmented pump therapy over 4 months in children with type 1 diabetes aged 1 to 7 years in the home setting.
**Short Title**	The artificial pancreas in very young children with T1D (KidsAP02)
**Sponsor name**	University of Cambridge & Cambridge University Hospitals NHS Foundation Trust
**Medical condition or disease under investigation**	Type 1 diabetes
**Study Phase**	Outcome study
**Purpose of clinical trial**	To determine whether 24/7 automated hybrid closed loop will improve glucose control as measured by time within the target range compared with sensor augmented pump therapy in very young children with T1D.
**Study objectives**	The study objective is to evaluate the safety, efficacy and utility of automated hybrid closed loop glucose control in very young children with type 1 diabetes.<br><br>1. EFFICACY: The objective is to assess the ability of a hybrid closed loop system to maintain CGM glucose levels within the target range of 3.9 to 10 mmol/l (70 to 180 mg/dl) in comparison with sensor augmented pump therapy in very young children with type 1 diabetes.<br><br>2. SAFETY: The objective is to evaluate the safety of closed loop glucose control compared with sensor augmented pump therapy in terms of episodes and severity of hypoglycaemia, frequency of diabetic ketoacidosis (DKA) and nature and severity of other adverse events.<br><br>3. UTILITY: The objective is to determine the acceptability and duration of use of the closed loop system in this population.<br><br>4. HUMAN FACTORS: The objective is to assess emotional and behavioural characteristics of participants and parents/guardians and their

Fuchs J, *et al. BMJ Open* 2021; 11:e042790. doi: 10.1136/bmjopen-2020-042790

	response to the closed loop system and clinical trial using validated surveys, actigraphy and semi-structured qualitative interviews.<br><br>5. HEALTH ECONOMICS: The objective is to perform a cost utility analysis to inform reimbursement decision-making.
**Study Design**	The study adopts an open-label, multi-centre, multi-national, randomised, two-period crossover study design contrasting closed loop glucose control and sensor augmented pump therapy in very young children with type 1 diabetes in the home setting. Two intervention periods will last 4 months each with 1 to 4 weeks washout period. The order of the two interventions will be random.
**Study Endpoints**	The primary endpoint:<br>The primary endpoint is the between group difference in time spent with sensor glucose levels between 3.9 to 10.0 mmol/l (70 to 180 mg/dl) during the 4 months intervention period.<br><br>Other key endpoints*<br>• Time spent above target glucose (10.0 mmol/l) (180 mg/dl)<br>• HbA1c<br>• Average of glucose levels<br>• Time spent below target glucose (3.9 mmol/l) (70 mg/dl)<br><br>Secondary endpoints* include the following:<br>• Standard deviation, and coefficient of variation of glucose levels<br>• Time with glucose levels <3.0 mmol/l (54 mg/dl)<br>• Time with glucose levels in significant hyperglycaemia (glucose levels > 16.7 mmol/l) (300 mg/dl)<br>• AUC of glucose below 3.5 mmol/l (63 mg/dl)<br>• BMI SDS<br>• Total, basal, and bolus insulin dose<br><br>* Endpoints regarding glucose levels will be based on sensor glucose data.
**Safety Evaluation**	Assessment of frequency and severity of hypoglycaemic episodes as defined by International Society for Pediatric and Adolescent Diabetes, frequency of diabetic ketoacidosis (DKA) and nature and severity of other adverse events.

**Utility Evaluation**	Assessment of the frequency and duration of use of the closed loop system.<br>• Percentage of closed loop operation<br>• Percentage of CGM availability
**Human factors assessment**	Emotional and behavioural characteristics of participants and family members and their response to the closed loop system and clinical trial will be assessed gathering both quantitative (validated surveys and actigraphy) and qualitative data (interviews).
**Health economic evaluation**	Cost utility analysis on the benefits of closed loop insulin delivery to inform reimbursement decision-making.
**Participating clinical centres**	1. Addenbrooke's Hospital, Cambridge University Hospital NHS Foundation Trust, Cambridge, UK<br>2. Leeds Teaching Hospitals NHS Trust, Leeds, UK<br>3. DECCP, Centre Hospitalier de Luxembourg, Grand Duché de Luxembourg<br>4. University of Leipzig, Leipzig, Germany<br>5. Medical University of Graz, Graz, Austria<br>6. Medical University of Innsbruck, Innsbruck, Austria<br>7. Medical University of Vienna, Vienna, Austria
**Sample Size**	72 participants randomised (8-12 participants per centre).
**Summary of eligibility criteria**	Key inclusion criteria:<br>1. Age between 1 and 7 years of age (inclusive)<br>2. Type 1 diabetes for at least 6 months<br>3. Insulin pump user for at least 3 months<br>4. Treated with rapid or ultra-rapid acting insulin analogue<br>5. Subject/carer is willing to perform at least 2 finger-prick blood glucose measurements per day<br>6. Screening HbA1c ≤ 11 % (97mmol/mol) based on analysis from local laboratory<br>7. Able to wear glucose sensor<br>8. Able to wear closed loop system 24/7<br>9. The subject/carer is willing to follow study specific instructions<br>10. The subject/carer is willing to upload pump and CGM data at regular intervals<br><br>Key exclusion criteria:

	1. Physical or psychological disease likely to interfere with normal conduct of the study<br>2. Untreated coeliac disease or thyroid disease<br>3. Current treatment with drugs known to interfere with glucose metabolism<br>4. Use of closed loop insulin delivery within the past two months<br>5. Known or suspected allergy to insulin<br>6. Carer's lack of reliable telephone facility for contact<br>7. Subject/carer's severe visual impairment<br>8. Subject/carer's severe hearing impairment<br>9. Medically documented allergy towards the adhesive (glue) of plasters or subject is unable to tolerate tape adhesive in the area of sensor placement<br>10. Serious skin diseases located at places of the body corresponding with sensor insertion sites<br>11. Sickle cell disease, haemoglobinopathy; or has received red blood cell transfusion or erythropoietin within 3 months prior to time of screening<br>12. Plan to receive red blood cell transfusion or erythropoietin over the course of study participation<br>13. Subject/carer not proficient in English (UK, Germany, Austria, Luxembourg) or German (Germany, Austria, Luxembourg) or French (Luxembourg)<br>Further exclusion criteria for Germany are listed in section 7.1.2.
**Maximum duration of study for a subject**	11 months
**Recruitment**	The subjects will be recruited through paediatric diabetes outpatient clinics at participating clinical centres (see above). Enrolment will target up to 80 (aiming for 8-12 participants per centre) to allow for dropouts during run-in.
**Consent**	Written informed consent will be obtained from all parents/guardians and written assent from older children before any study related activities.
**Baseline Assessment**	Eligible subjects will undergo a baseline assessment including a blood sample for the measurement of HbA1c. Questionnaires will be completed by parents/guardians.

**Pre-Study Training and Run-in**	Training sessions on the use of the study CGM and insulin pump will be provided by the research team. During a 2-4 week run-in period, subjects will use study CGM and insulin pump. For compliance and to assess the ability of the subject to use the study devices safely, at least 8 days of CGM data need to be recorded and safe use of study insulin pump demonstrated during the last 14 days of run-in period. The CGM data will also be used to assess baseline glucose control and may be used for treatment optimization as necessary.
**Competency Assessment**	Competency on the use of study pump and study CGM will be evaluated using a competency assessment tool developed by the research team. Training may be repeated if required.
**Randomisation**	Eligible subjects will be randomised using randomisation software to the initial use of automated hybrid closed loop glucose system or to sensor augmented pump therapy for 4 months with a 1 to 4 week washout period before crossing over to the other study arm.<br><br>A blood sample for HbA1c will be taken if screening and randomisation are >28 days apart.
**1. Automated day and night closed loop insulin delivery (intervention arm)**	Participants in the closed loop arm and their caregivers will receive an additional training session covering the use of the closed loop system provided by the research team prior to starting closed loop insulin delivery. During this 1-2 hour session, parents/guardians will operate the system under the supervision of the clinical research team. Competency on the use of closed loop system will be evaluated. Thereafter, subjects and their parents/guardians will use the hybrid closed loop system for 4 months at home.
**Crossover Assessment**	At the end of the first study arm, a blood sample for the measurement of HbA1c will be taken and weight and height will be measured. Validated surveys evaluating the impact of the devices employed on quality of life, psychosocial function, diabetes management and treatment satisfaction will be completed.<br><br>Parents/guardians will be invited to be interviewed to gather feedback on and reactions to their current treatment, the clinical trial, and quality of life changes.

Fuchs J, *et al. BMJ Open* 2021; 11:e042790. doi: 10.1136/bmjopen-2020-042790

**2. Sensor augmented pump therapy (control arm)**	Participants in the sensor augmented pump therapy arm and their caregivers will receive refresher training on key aspects of insulin pump therapy and CGM use.<br><br>Subjects and their parents/guardians will continue using sensor augmented pump therapy for 4 months at home.
**Study contacts**	Participants will be contacted 24h after starting each study arm to ensure there are no concerns regarding the study devices. In between study visits, participants will be contacted by the study team (email/phone) once monthly in order to record any adverse events, device deficiencies, and changes in insulin settings, other medical conditions and/or medication.<br><br>In case of any problems related to the technical device or diabetes management such as hypo- or hyperglycaemia, subjects will be able to contact a 24-hour telephone helpline to the local research team at any time. The local research team will have access to central 24 hour advice on technical issues.
**End of study assessments**	A blood sample will be taken for measurement of HbA1c at the end of the study. Height and weight will be recorded. Study devices will be downloaded and returned. Participants will resume usual care using their pre-study insulin pump. Validated surveys evaluating the impact of the devices employed on quality of life, psychosocial function and diabetes management and treatment satisfaction will be completed.<br><br>Parents/guardians will be invited to participate in a sleep sub-study prior to the final visit.<br><br>Parents/guardians will be invited to be interviewed to gather feedback on and reactions to their current treatment, the clinical trial, and quality of life changes.
**Procedures for safety monitoring during trial**	Standard operating procedures for monitoring and reporting of all adverse events will be in place, including serious adverse events (SAE), serious adverse device effects (SADE) and specific adverse events (AE) such as severe hypoglycaemia.<br><br>A data safety and monitoring board (DSMB) will be informed of all serious adverse events and any unanticipated serious adverse device effects that occur during the study and will review compiled adverse event data at periodic intervals.
**Criteria for withdrawal of subjects on safety grounds**	A subject/guardian may terminate participation in the study at any time without necessarily giving a reason

Fuchs J, *et al. BMJ Open* 2021; 11:e042790. doi: 10.1136/bmjopen-2020-042790

	and without any personal disadvantage. An investigator can stop the participation of a subject after consideration of the benefit/risk ratio. Possible reasons are:<br>• Serious adverse events<br>• Non-compliance<br>• Serious protocol violation<br>• Decision by the investigator, or the sponsor, that termination is in the subject's best medical interest<br>• Allergic reaction to insulin

3 Summary

The suggested clinical trial is part of the KidsAP project funded by the European Commission's Horizon 2020 Framework Programme with additional funding by JDRF. The project evaluates the use of the Artificial Pancreas (or closed loop system) in very young children with type 1 diabetes (T1D) aged 1 to 7 years. The suggested trial is an outcome study to determine whether 24/7 automated closed loop glucose control will improve glucose control as measured by time in range compared to sensor augmented pump therapy.

This is an open-label, multi-centre, multi-national, randomised, two period, cross-over design study, contrasting a 4 month period during which glucose levels will be controlled either by a closed loop system (intervention group) or by sensor augmented pump therapy (control group). The order of the two interventions will be random.

A total of up to 80 young children aged 1 to 7 years with T1D on insulin pump therapy (aiming for 72 randomised subjects) will be recruited through paediatric outpatient diabetes clinics of the investigation centres.

Prior to the use of study devices, participants and parents/guardians will receive appropriate training by the research team on the safe use of the study pump and CGM device, and the hybrid closed loop insulin delivery system. Carers at nursey/school may also receive training by the study team if required.

During the closed loop study arm, subjects and parents/guardians will use the closed loop system for 4 months under free-living conditions in their home and nursery/school environment. During the control study arm, subjects and parents/guardians will use sensor augmented pump therapy for 4 months under free-living conditions in their home and nursery/school environment. All subjects will have regular contact with the study team during the home study phase including 24/7 telephone support.

The primary endpoint is time spent in target range, between 3.9 and 10.0 mmol/l as recorded by CGM. Secondary outcomes are the time spent with glucose levels above and below target, as recorded by CGM, and other CGM-based metrics. Safety evaluation comprises assessment of the frequency and severity of hypoglycaemic episodes and diabetic ketoacidosis (DKA).

Fuchs J, *et al. BMJ Open* 2021; 11:e042790. doi: 10.1136/bmjopen-2020-042790

4 Background

4.1 Introduction

Type 1 diabetes is associated with life-long dependency on insulin administration and is caused by immune-mediated destruction of the pancreatic beta cells in genetically predisposed individuals (1). Type 1 diabetes (T1D) is one of the commonest chronic conditions in childhood, and the incidence is increasing worldwide (2) with an estimated overall annual rate of increase of approximately 3%, including in the youngest age group (3). Achievement of tight glycaemic control in T1D is limited by hypoglycaemia, contributing to the majority of youths failing to meet treatment guidelines for target glycated haemoglobin (HbA1c) below 7.5% (58.5 mmol/mol) (4).

The continuing emergence of innovative technologies has shaped and changed management and care in T1D over the past decades. The use of insulin pumps is increasing, particularly in the paediatric population with 50% to 79% of those below the age of 6 years using pumps (5, 6). Real-time continuous glucose monitoring enables greater understanding of glucose excursions, provides low and high glucose alarms, and facilitates more responsive insulin dose adjustments (7) but provides little biochemical benefit in young children although it is well accepted by parents (8).

The Artificial Pancreas is an emerging technology promising to transform management of T1D (9-11). The Artificial Pancreas gradually increases and decreases subcutaneous insulin delivery according to real-time sensor glucose levels, combining glucose sensor, insulin pump and a control algorithm, to achieve as much as possible functionality of a healthy pancreas. In the last five years, outpatient studies by University of Cambridge and collaborators (12-16), University of Virginia and collaborators (17-19), Boston University (20, 21), Medtronic 670G pump and prototypes (22), and MD-Logic group (23-25) showed feasibility and some efficacy of outpatient closed loop use.

The case for the Artificial Pancreas use in young children is supported by diabetes registries (4, 26, 27) which indicate that glycaemic control in pre-school children is often sub-optimal, even when applying continuous glucose monitoring (8, 28) with only between 22% and 56% achieving the recommended HbA1c below 7.5% (58.5mmol/mol) (10). A life-long exposure to hyperglycaemia may lead to an accentuated risk of late micro and macrovascular complications. An early onset of diabetes has also been more strongly associated with impaired cognitive function (29-32) and reduced school achievements (33), with recent evidence of dysglycaemia-related anatomical brain changes in young children with type 1 diabetes (34, 35). High

Fuchs J, *et al. BMJ Open* 2021; 11:e042790. doi: 10.1136/bmjopen-2020-042790

insulin sensitivity as well as unpredictable food intake and physical activity, and day-to-day variable insulin requirements (36) complicate insulin dose adjustments. Reductions in HbA1c using existing therapies are associated with an increased risk of hypoglycaemia, feared by children and their caregivers (37). Educational interventions in youth are failing (38) and immunotherapy struggles in clinical translation (39). CGM has little impact (28). Novel treatment strategies are needed to improve outcomes in this vulnerable and underserved population.

4.2 Closed Loop Insulin Delivery

The development of a closed loop system combines glucose monitoring with computer-based algorithm informed insulin delivery. The vital component of such a system, also known as an artificial pancreas (AP), is a computer-based algorithm. The role of the control algorithm is to translate, in real-time, the information it receives from the CGM and to compute the amount of insulin to be delivered by the pump. The other components include a real-time continuous glucose monitor and an infusion pump to titrate and deliver insulin (9).

Automatic suspension of insulin delivery by the pump when a predefined glucose level is reached (threshold suspend insulin pump therapy) represents the simplest form of closed loop insulin delivery. Such a system (Veo (non-US) or Medtronic 530G (US) insulin pump coupled with Minilink sensor (Medtronic Minimed, Northridge, CA, USA) which stops insulin delivery for up to 2 hours is currently commercially available (40-43). In comparison to threshold suspension, hypoglycaemia-prediction algorithms and automatic pump suspension of predictive log glucose suspension (PLGS) systems enable insulin delivery to be suspended when hypoglycaemia is predicted (44). The predictive low glucose management (PLGM) function of the MiniMed 640G (Medtronic Diabetes, Northridge, CA, USA) insulin pump was introduced into clinical practice in Australia and Europe early 2015. A slightly revised version was recently approved by the FDA in the US for the treatment of people with diabetes sixteen years of age and older (MiniMed 630G; Medtronic Diabetes, Northridge, CA, USA). Since insulin is not delivered in an automated fashion there is no risk of system-induced hypoglycaemia. The risk of consecutive rebound hyperglycaemia and ketonaemia after a temporary suspension of insulin administration is not negligible (45), but only mild rebound hyperglycaemia and no episodes of diabetic ketoacidosis following threshold suspension activation have been reported (43, 46). While threshold suspend insulin delivery systems and predictive low glucose suspension systems might reduce the risk and severity of hypoglycaemic events, neither system mitigates against higher glycaemic levels.

Fuchs J, et al. BMJ Open 2021; 11:e042790. doi: 10.1136/bmjopen-2020-042790

Closed loop approaches beyond insulin suspension have been successfully evaluated in children and adolescents in controlled laboratory studies (23, 47, 48) and in home settings (12, 15, 16, 49). Investigations in adults have also been conducted (13, 14, 16). In these studies more advanced control algorithms were used which autonomously and continually increased and decreased subcutaneous insulin delivery based on real-time sensor glucose levels. The results demonstrated improved glucose control and reduced risk of hypoglycaemia events. Psychosocial assessments supported acceptability and positive impact of this novel therapeutic approach among children/adolescents and carers (50). Evaluations during home, free living conditions have been limited to 3 months (16).

4.3 Closed Loop Research in Cambridge

The University of Cambridge and collaborators have a considerable track record investigating closed loop glucose control in young children, older children, adolescents, adults, and pregnant women with type 1 diabetes (12, 15, 16, 51, 52). Since 2012, the University of Cambridge with collaborators have enrolled over 180 subjects in RCTs of free-living closed loop home conditions lasting 1 week to 2 years focusing on young people.

4.3.1 Closed Loop Prototypes

The University of Cambridge developed several home prototypes differing in computer algorithm hosting device, connectivity to the CGM receiver, and remote data upload (details not shown) but using the same Cambridge control algorithm. The previous FlorenceD2A Android-based system is shown in Figure 1.

Dana pump was selected due to its best-in-class remote functions enabling reliable closed loop operation and Navigator II CGM system was chosen because of its high accuracy. These systems used the **Cambridge model predictive control** algorithm including an interacting multiple model strategy with meal- and exercise-announcement. The algorithm directs insulin delivery between meals and overnight with prandial insulin delivered using a standard bolus calculator. Key characteristics of the control algorithm are its ease of setup (body weight and total daily dose), adaptability, a safety layer, and extensive clinical data to support safety and efficacy.

Fuchs J, *et al. BMJ Open* 2021; 11:e042790. doi: 10.1136/bmjopen-2020-042790

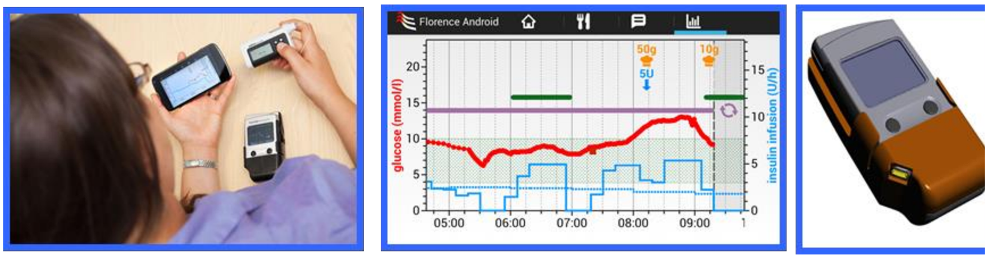

Figure 1 FlorenceD2A closed loop system using Android phone, Navigator II (Abbott Diabetes Care, USA), and Dana insulin pump (Sooil, South Korea) with wireless connection to Navigator II using translator (Triteq, UK) and remote cloud connectivity (left – system components, middle – combined graph shown on the phone, right – Navigator inserted in the translator).

Preliminary Data with the Cambridge Control Algorithm

In 2012, the University of Cambridge and collaborators initiated a series of RCTs of
4.3.2 free-living closed loop studies (Table 1). Characteristics of these home studies include no remote monitoring or close supervision, unlike most other transitional, diabetes camp, or outpatient studies (18, 21, 24, 25). Closed loop was used under free-living conditions at school, work, and over holidays.

Table 1 Free-living home closed loop studies using Cambridge control algorithm.

**Study acronym**	**Closed loop system**	**Design & closed loop follow-up**	**T1D population**	**Status**	**Key Refs**
APCam06	FlorenceD2**	Crossover RCT, overnight CL*, 3 weeks	n=16 10-18 years	Study completed	(12)
Angela03	FlorenceD2	Multicentre crossover RCT, overnight CL, 4 wks	n=24 adults	Study completed	(13)
APhome02	FlorenceD2	Multinational crossover RCT, 24/7 CL, 1 week	n=17 adults	Study completed	(14)
APCam08	FlorenceD2W †	Multicentre crossover RCT, o'night CL, 12 wks	n=24 6-18 years	Study completed	(16)
Dan04 Phase 1	FlorenceD2A	Crossover RCT, 24/7 CL, 1 week	n=12 12-18 years	Study completed	(15)
Dan04 Phase 2	FlorenceD2A	Crossover RCT, 24/7 CL, 3 weeks	n=12 12-18 years	Study completed	(53)
Aphome04	FlorenceD2A	Multinational crossover RCT, 24/7 CL, 12 weeks	n=31 adults	Study completed	(16)
APCam11	FlorenceM	Multinational parallel RCT, 24/7 CL, 12 weeks	n=84 6yrs-adults	Study completed	-
CLOuD	FlorenceM	Multicentre parallel RCT, 24/7 CL; 2 years	n=96 10-18 years	Started Q1 2017	-
KidsAP01	FlorenceM	Multinational crossover RCT, 24/7 CL, 2x3 weeks	n=24 1-7yrs	Study completed	-

*CL – closed loop ** Comprising ultramobile Windows laptop with wired connection to Navigator; no remote connectivity † Windows tablet with wired connection to Navigator II; no remote connectivity.

APCam06 and Angela03. Overnight closed loop insulin delivery over 3 to 4 weeks reduced overnight glucose by mean 0.8 mmol/l in 16 adolescents (HbA1c 8.0% [64 mmol/mol]) in a single centre randomised study (p<0.001) (12) and 24 adults (HbA1c 8.1% [65 mmol/mol]) in a multicentre randomised control study (p<0.005) (13). After pooling data (total 850 closed loop nights) and adopting intention to treat analysis, the proportion of time spent overnight in hypoglycaemia below 3.9 mmol/l was reduced from median 2.9% to median 1.9% (p=0.014), mean overnight glucose reduced (p<0.001), and time spent in the target glucose range increased (p<0.001) (Table 2) (54).

Table 2 Outcomes during overnight closed loop and sensor augmented pump therapy in the home setting over 3 to 4 weeks combining APCam06 and Angela03

	**Closed loop (n=40)**	**Control (n=40)**	**P value**
Time spent at glucose level (%)			
3.9 to 8.0 mmol/l	59.2±11.5	40.7±13.4	<0.001
> 8 mmol/l	37.9±12.4	53.8±17.0	0.001
< 3.9 mmol/l	1.9 (0.7, 3.5)	2.9 (1.0, 6.4)	0.014
Mean glucose (mmol/l)	7.9±0.9	8.7±1.4	<0.001
Total daily insulin delivery (U)	40.3 (32.9, 52.6)	39.4 (32.8, 55.8)	0.84

Data shown are mean ± SD or median (IQR)

APhome02. A multicentre multinational crossover RCT in 17 adults (HbA1c 7.6% [59 mmol/mol]) involving University of Cambridge and Medical University of Graz and investigating day-and-night free-living closed loop over one week documented reduced 24hour glucose by mean 0.7 mmol/l (p=0.027) while time spent in hypoglycaemia below 3.9 mmol/l was unchanged (p=0.34) (Table 3). The proportion of time spent with glucose in the target range from 3.9 to 10 mmol/l increased from median 62% to median 75% (p=0.005) with a trend towards reduced total daily insulin dose from mean 45 U/day to 39 U/day (p=0.11). This suggests that insulin can be more efficiently and sparingly titrated by an automated control algorithm compared to pre-programmed pump delivery (14).

Table 3 Glucose control during 24/7 closed loop and sensor augmented pump therapy over seven day free-living home use in 17 adults with type 1 diabetes (APhome02 study).

	**Closed loop (n=17)**	**Control (n=17)**	**P value**
Time spent at glucose (%)			
3.9 to 10.0 mmol/l	75 (61, 79)	62 (53, 70)	0.005
> 10 mmol/l	22 (17, 32)	31 (24, 41)	0.013
< 3.9 mmol/l	3.7 (2.2, 7.9)	5.0 (2.3, 8.5)	0.339

Fuchs J, *et al. BMJ Open* 2021; 11:e042790. doi: 10.1136/bmjopen-2020-042790

Mean glucose (mmol/l)	8.1 ± 1.0	8.8 ± 1.0	0.027
Total daily insulin (U/day)	39.1 (34.7, 45.7)	44.7 (36.3, 51.0)	0.109

Dan04 Phase 1. In an open-label, crossover randomised study the University of Cambridge evaluated feasibility, safety and efficacy of 24/7 closed loop under free-living conditions. In random order, 12 children and adolescents with T1D aged 12 to 18 years (age 15.4±2.6 years; HbA1c 8.3 ± 0.9%) underwent two 7 day periods of sensor augmented insulin pump therapy or closed loop.

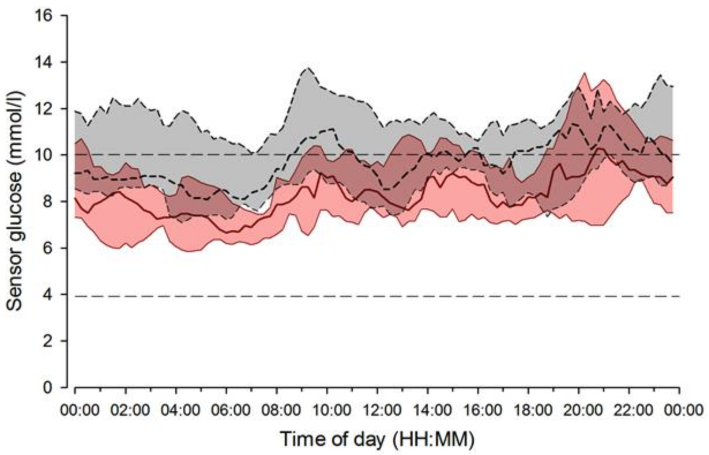

Figure 2 Sensor glucose during 24/7 closed loop (pink area) and sensor augmented pump therapy (grey area) in 12 young people aged 12 to 18 years (n=12) participating in Dan04 phase 1 study [median (IQR) range].

Time when sensor glucose was in target range from 3.9 to 10 mmol/l, was significantly higher during closed loop compared to SAP (p<0.001, Table 4 and Figure 2). Mean glucose (p=0.03) and time spent above target (p=0.005) were lower during closed loop, without changing total daily insulin dose (15).

Table 4 Glucose control during 24/7 closed loop and sensor augmented pump therapy over seven day home use in 12 youth aged 10 to 18 years with type 1 diabetes (Dan04 phase 1 study).

	Closed loop (n=12)	Control (n=12)	P value
Time spent at glucose (%)			
3.9 to 10.0 mmol/l	72 (59, 77)	53 (46, 59)	<0.001
> 10 mmol/l	26 (21, 35)	43 (38, 52)	0.005
< 3.9 mmol/l	2.9 (1.8, 4.8)	1.7 (0.9, 5.1)	0.87
Mean glucose (mmol/l)	8.7 ± 1.1	10.2 ± 1.3	0.03
Total daily insulin (U/day)	57.3 (45.6, 65.2)	56.6 (44.7, 61.3)	0.55

Dan04 Phase 2. In an open-label, crossover randomised study the University of Cambridge evaluated feasibility, safety and efficacy of 24/7 closed loop under free-

Fuchs J, et al. BMJ Open 2021; 11:e042790. doi: 10.1136/bmjopen-2020-042790

living conditions. In random order, 12 children and adolescents with suboptimally controlled T1D aged 12 to 18 years (age 14.6±3.1 years; HbA1c 69±8 mmol/mol [8.5±0.7%]; duration of diabetes 7.8±3.5 years; mean±SD) underwent two 21 day periods of sensor augmented insulin pump therapy or closed loop.

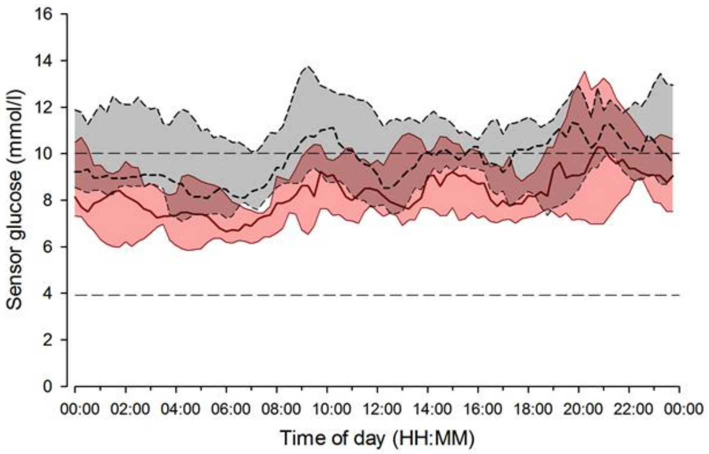

Figure 3 Sensor glucose during 24/7 closed loop (pink area) and sensor augmented pump therapy (grey area) in 12 young people aged 12 to 18 years (n=12) participating in Dan04 phase 2 study [median (IQR) range].

Time when sensor glucose was in target range from 3.9 to 10 mmol/l, was significantly higher during closed loop compared to SAP (p<0.001, Table 5 and Figure 3). Mean glucose (p=0.001) and time spent above target (p<0.001) were lower during closed loop, with a slight increase in total daily insulin dose (p=0.006) (53).

Table 5 Glucose control during 24/7 closed loop and sensor augmented pump therapy over seven day home use in 12 youth aged 10 to 18 years with type 1 diabetes (Dan04 phase 2 study).

	**Closed loop (n=12)**	**Control (n=12)**	**P value**
Time spent at glucose (%)			
3.9 to 10.0 mmol/l	66.6±7.9	47.7±14.4	<0.001
> 10 mmol/l	29.7±9.2	49.1±16.5	<0.001
< 3.9 mmol/l	4.3 (1.4 to 5.2)	2.4 (0.3 to 5.7)	0.33
Mean glucose (mmol/l)	8.7±0.9	10.5±1.8	0.001
Total daily insulin (U/day)	53.5 (39.5 to 72.1)	51.5 (37.6 to 64.3)	0.006

APCam08. The University of Cambridge and Leeds Teaching Hospital completed a multicentre crossover RCT comparing 12 week overnight closed loop with 12 week sensor augmented pump therapy in 25 children and adolescents aged 6 to 18 years. The intention to treat analysis (Table 6) documents reduced mean overnight glucose (p<0.003), increased time spent in target range (p<0.001) (16).

Table 6 Glucose control during overnight closed loop and sensor augmented pump therapy over 12 weeks free-living home use in young people aged 6 to 18 years with type 1 diabetes (APCam08 study).

	Closed loop (n=25)	Control (n=24)	P value
Time spent at glucose (%)			
3.9 to 8.0 mmol/l	60 ± 12	34 ± 11	<0.001
> 8 mmol/l	37 ± 12	61 ± 13	<0.001
< 3.9 mmol/l	2.2 (1.8, 4.3)	3.5 (1.2, 5.9)	0.7
Mean overnight glucose (mmol/l)	8.1 ± 1.2	9.8 ± 1.6	<0.001
Total daily insulin (U/day)	41.4 ± 20.3	40.9 ± 20.6	0.8

APhome04. In a 12-week open-label prospective multinational randomised crossover study involving the University of Cambridge and Medical University of Graz, we analysed data from adults with type 1 diabetes on insulin pump therapy (18 male, age 40.0±9.4 years, HbA1c 8.5±0.7%; duration of diabetes 20.9±9.3 years) who underwent two 12-week periods of SAP and 24/7 closed loop (16).

The proportion of time when sensor glucose was in target range between 3.9 and 10.0 mmol/l was increased during closed loop compared to SAP (p<0.001; Table 7).

Table 7 Glucose control during 24/7 closed loop and sensor augmented pump therapy over 12 weeks free-living home use in adults with type 1 diabetes (APhome04 study).

	Closed loop (n=32)	Control (n=33)	P value
Time spent at glucose (%)	67.7 ± 10.6	56.8 ± 14.2	<0.001
3.9 to 10.0 mmol/l			
> 10 mmol/l	29.2 ± 11.4	38.9 ± 16.6	<0.001
< 3.9 mmol/l	2.9 (1.4, 4.5)	3.0 (1.8, 6.1)	0.02
Mean glucose (mmol/l)	8.7 ± 1.1	9.3 ± 1.6	<0.001
HbA1c (%)	7.3 ± 0.8	7.6 ± 1.1	0.002
Total daily insulin (U/day)	48.8 ± 16.1	48.1 ± 15.4	0.6

HbA1c, mean glucose and time spent above target range were significantly reduced during closed loop, while time spent below target was low and comparable during both interventions. Hypoglycaemia exposure measured by AUC <3.5 mmol/l was reduced during closed loop (p=0.004). Reduction in mean glucose and time spent above target range during closed loop was brought about without changing the total daily insulin delivery (p=0.6).

Fuchs J, *et al. BMJ Open* 2021; 11:e042790. doi: 10.1136/bmjopen-2020-042790

KidsAP01. In an open-label, multinational, randomised crossover study involving KidsAP Consortium we analysed data from 23 young children aged 1 to 7 years on insulin pump therapy (age 5 (3 to 6) years, median (IQR): median HbA1c 7.4±0.7%, mean±SD: duration of diabetes 3.1 ±1.7 years) who underwent two 21-day periods of unrestricted living comparing CL with diluted insulin aspart (U20) and CL with standard strength insulin aspart (U100) in random order.

The proportion of time that sensor glucose was in the target range between 3.9 and 10mmol/l (primary endpoint) was similar between interventions (72±8% vs. 70±7%; CL with diluted U20 insulin vs. CL with standard strength U100 insulin; p=0.14: Table 8).

Table 8 Glucose control during 24/7 closed loop with diluted insulin aspart (U20) and CL with standard strength insulin aspart (U100) over two 21-day periods of free-living home use in 23 children with type 1 diabetes aged 1 to 7 years (KidsAP01 Study).

	**Diluted (n=23)**	**Non diluted (n=23)**	**P value**
% Time in Range 3.9-10.0 mmol/L	72% ± 8%	70% ± 7%	0.14
% Time >10.0 mmol/L	23% ± 9%	25% ± 7%	0.21
% Time <3.9 mmol/L	4.5% ± 1.7%	4.7% ± 1.5%	0.46
Mean Glucose (mmol/L)	8.0 ± 0.8	8.2 ± 0.6	0.12
Total Insulin (U/day)	17.3 ± 5.6	18.4 ± 6.6	0.09

Data shown are mean ± SD

There was no difference between interventions either in mean sensor glucose (8.0±0.8mmol/l vs. 8.2±0.6mmol/l; p=0.12) or sensor glucose variability (SD 3.1±0.5mmol/l vs. 3.3±0.4mmol/l; p=0.14). The proportion of time when sensor glucose was below 3.9mmol/l (4.5±1.7% vs. 4.7±1.5%; p=0.46) or below 2.8mmol/l (0.6±0.5% vs. 0.6±0.4%; p=0.98) was comparable. Total daily insulin dose did not differ between interventions (17.3±5.6U/day vs. 18.4±6.6U/day; p=0.09).

No CL-related severe hypoglycaemia or ketoacidosis occurred.

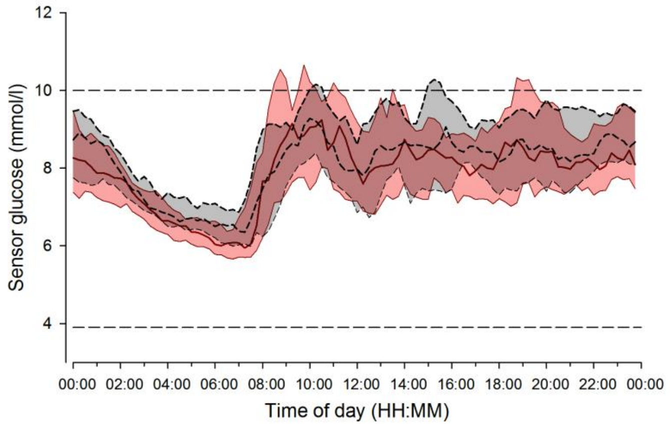

Figure 4. Sensor glucose during 24/7 closed loop with diluted insulin aspart (U20) (pink area) and CL with standard strength insulin aspart (U100) (grey area) over two 21-day periods of free-living home use in 23 children with type 1 diabetes aged 1 to 7 years participating KidsAP01 study [median (IQR) range].

Psychosocial outcomes. We examined parental attitudes to overnight closed loop in families with children and young people with type 1 diabetes (55). The development of closed loop to manage diabetes was welcomed by all parents (100%). All parents were ready to respond to additional alarms at night with 90% parents not worried about their child's overnight insulin delivery being controlled by a computer (55). These positive attitudes towards closed loop were confirmed in semi-structured interviews with 15 adolescents aged 12 to 18 years and 13 parents participating in a three week long home overnight closed loop APCam06 study (50) (47). Similar observations were made in 24 adults undergoing home overnight closed loop (56).

4.4 KidsAP Consortium

In December 2016, the European Union granted funding for the KidsAP project, a consortium of European academic medical centres, biotechnology companies and industrial partners to carry out artificial pancreas/closed loop research in the paediatric population outside clinical research centres. The ultimate goal of the KidsAP project is to assess the ability of the artificial pancreas to improve glucose control in the most vulnerable population with type 1 diabetes, children aged 1 to 7 years. A pilot study (KidsAP01) assessed the efficacy and safety of closed loop insulin delivery using diluted insulin in comparison with closed loop with non-diluted insulin over 21 days in children with type 1 diabetes aged 1 to 7 years in the home setting. It was demonstrated that diluted insulin during CL does not provide additional benefits compared to standard strength insulin.

The results of the KidsAP01 study have informed the design of this outcome study.

4.5 FlorenceX Closed Loop System to be used in the Present Study

In the present study, we will use the FlorenceX closed loop system comprising:

- Dana insulin pump (Diabecare, Sooil, Seoul, South Korea)
- Dexcom G6 real-time CGM sensor (Dexcom, Northridge, CA, USA)
- An Android smartphone hosting FlorenceX Application with the Cambridge model predictive control algorithm and communicating wirelessly with the insulin pump
- Cloud upload system to monitor CGM/insulin data.

An overview of this proposed automated closed loop system is given in Figure 4.

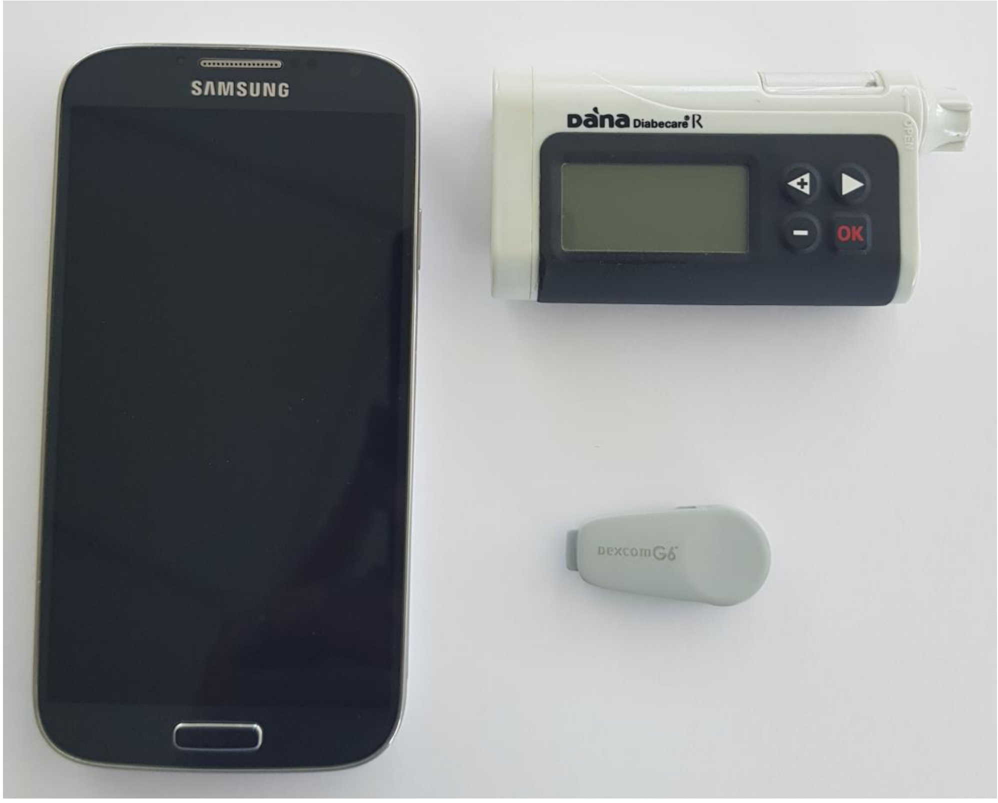

Figure 4 FlorenceX comprises Samsung Galaxy phone (or similar) running Cambridge control algorithm, Dana insulin pump (Sooil), G6 real-time CGM sensor (Dexcom).

4.6 Rationale for the present study

Despite advances in insulin pump and sensor technology, the majority of small children with type 1 diabetes are still unable to achieve optimal glycaemic control. Application of closed loop insulin delivery systems in a range of populations and settings, including children and adolescents in home settings, has been shown to improve glycaemic

control, and to reduce the burden of hypoglycaemia (15, 23, 49, 57, 58). Performance of closed loop systems in small children in home settings is yet to be determined.

In the present study we will compare closed loop insulin delivery with sensor augmented pump therapy in very young children with T1D. We hypothesize that closed loop glucose control will give increased time in range compared to sensor augmented pump therapy. This study builds on previous and on-going studies of closed loop systems that have been performed in Cambridge in children and adolescents with T1D in clinical research facilities and in the home setting.

The ultimate goal of the KidsAP project is to assess the ability of the artificial pancreas to improve glucose control and health outcomes in the most vulnerable population with type 1 diabetes: children aged 1 to 7 years.

5 Objectives

5.1 Efficacy

The objective is to assess the ability of day-and-night hybrid closed loop glucose control to maintain CGM glucose levels within the target range of 3.9 to 10 mmol/l (70 to 180 mg/dl) in comparison to sensor augmented pump therapy in young children with type 1 diabetes.

5.2 Safety

The objective is to evaluate the safety of day-and-night hybrid closed loop glucose control, in terms of frequency and severity of hypoglycaemia, as defined by International Society for Pediatric and Adolescent Diabetes, frequency of diabetic ketoacidosis (DKA), and nature and severity of other adverse events.

5.3 Utility

The objective is to determine the acceptability, duration and frequency of use of the closed loop system in this population. A series of questionnaires will be given to parents/guardians at the end of each intervention arm.

Fuchs J, *et al. BMJ Open* 2021; 11:e042790. doi: 10.1136/bmjopen-2020-042790

5.4 Human Factors

The objective is to assess emotional and behavioural characteristics of participating subjects and family members and their response to the closed loop system and clinical trial using quantitative (validated surveys and actigraphy) and qualitative data (interviews).

5.5 Health Economics

The objective is to perform a cost utility analysis on the benefits of closed loop insulin delivery to inform reimbursement decision-making.

6 Study Design

The study adopts an open-label, multi-centre, multi-national, randomised, two-period crossover design contrasting closed loop glucose control to sensor augmented pump therapy in young children with type 1 diabetes in the home setting. Two intervention periods will last 4 months each with 1 to 4 weeks washout period in between. The order of the two interventions will be random.

It is expected that up to 80 young children with type 1 diabetes will be recruited, aiming for 72 randomised subjects.

The study flow chart is outlined in Figure 5.

Figure 5 Study flow chart.

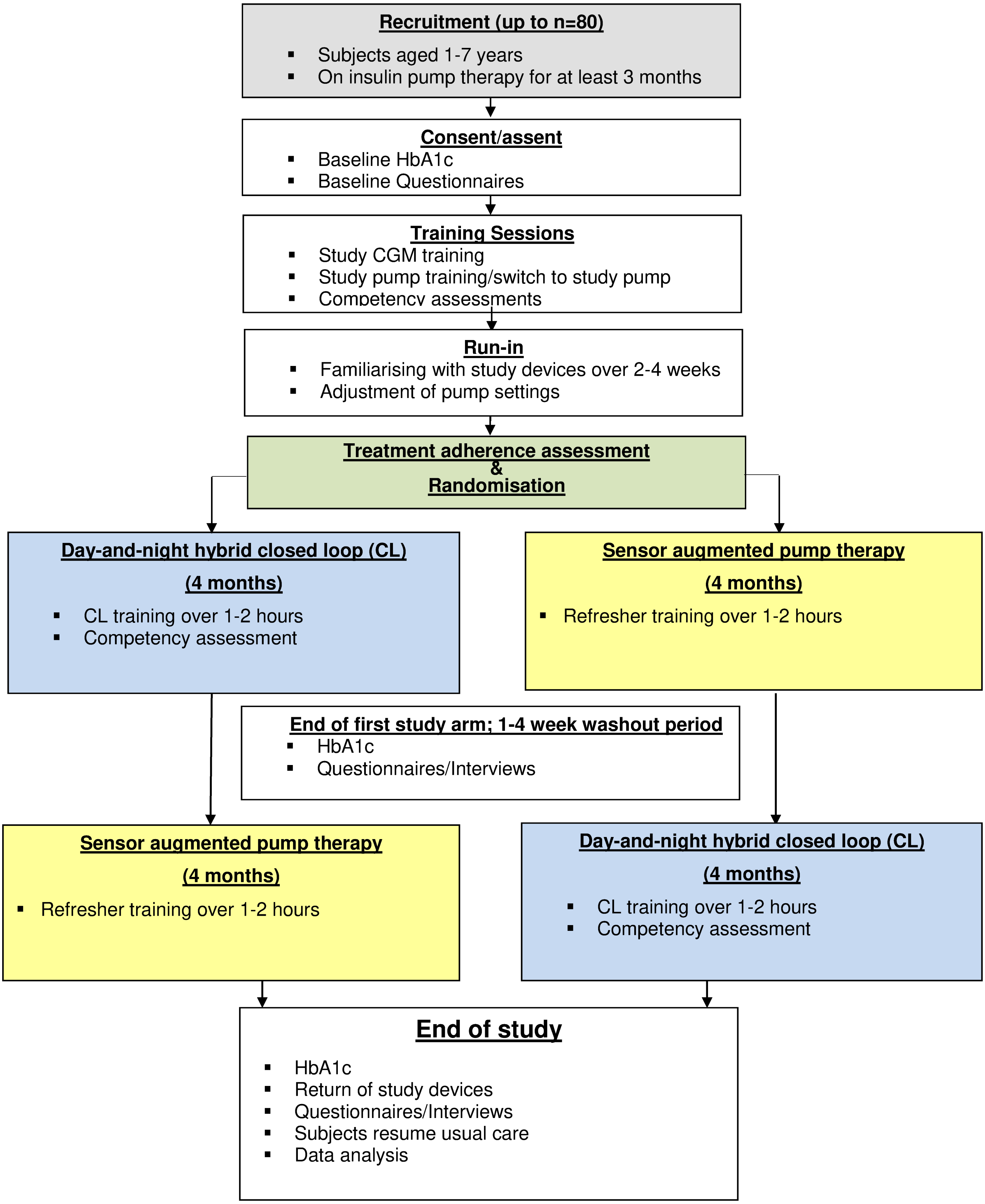

Fuchs J, *et al. BMJ Open* 2021; 11:e042790. doi: 10.1136/bmjopen-2020-042790

7 Study Subjects

7.1 Study Population

Young children aged 1 to 7 years with type 1 diabetes on insulin pump therapy will be recruited.

Inclusion criteria

1. Age between 1 and 7 years (inclusive) (Luxembourg and Austria)
2. Age between 2 and 7 years (inclusive) (Germany and UK)

7.1.1

3. Type 1 diabetes as defined by WHO for at least 6 months
   [WHO definition: 'The aetiological type named type 1 encompasses the majority of cases which are primarily due to beta-cell destruction, and are prone to ketoacidosis. Type 1 includes those cases attributable to an autoimmune process, as well as those with beta-cell destruction for which neither an aetiology nor a pathogenesis is known (idiopathic). It does not include those forms of beta-cell destruction or failure to which specific causes can be assigned (e.g. cystic fibrosis, mitochondrial defects, etc.).']
4. Insulin pump user (with or without continuous glucose monitoring or flash glucose monitoring system) for at least 3 months, with subject/carer good knowledge of insulin self-adjustment as judged by the investigator
5. Treated with rapid or ultra-rapid acting insulin analogue
6. Subject/carer is willing to perform regular finger-prick blood glucose monitoring, with at least 2 blood glucose measurements taken every day
7. Screening HbA1c ≤ 11% (97mmol/mol) on analysis from local laboratory
8. Able to wear glucose sensor
9. Able to wear closed loop system 24/7 during intervention arm
10. The subject/carer is willing to follow study specific instructions

7.1.2

11. The subject/carer is willing to upload pump and CGM data at regular intervals

Exclusion criteria

1. Physical or psychological disease likely to interfere with the normal conduct of the study and interpretation of the study results as judged by the investigator
2. Untreated coeliac disease or thyroid disease based on local investigations prior to study enrolment
3. Current treatment with drugs known to interfere with glucose metabolism, e.g. systemic corticosteroids
4. Use of closed loop insulin delivery within the past 2 months
5. Known or suspected allergy to insulin
6. Carer's lack of reliable telephone facility for contact

Fuchs J, *et al. BMJ Open* 2021; 11:e042790. doi: 10.1136/bmjopen-2020-042790

7. Subject/carer's severe visual impairment
8. Subject/carer's severe hearing impairment
9. Medically documented allergy towards the adhesive (glue) of plasters or subject is unable to tolerate tape adhesive in the area of sensor placement
10. Serious skin diseases (e.g. psoriasis vulgaris, bacterial skin diseases) located in parts of the body which could potentially be used for localisation of the glucose sensor)
11. Sickle cell disease, haemoglobinopathy; or has received red blood cell transfusion or erythropoietin within 3 months prior to time of screening
12. Plan to receive red blood cell transfusion or erythropoietin over the course of study participation
13. Subject/carer not proficient in English (UK, Germany, Austria, Luxembourg) or German (Germany, Austria, Luxembourg) or French (Luxembourg)

Additional exclusion criteria - Germany only

14. Known microvascular diabetes complications (retinopathy, renal disease, neuropathy)
15. Eating disorders
16. Psychiatric diseases of the parents that would possibly interfere with the ability to comply to study procedures
17. Major needle phobia that would complicate to wear pump catheter and sensor
18. Congenital malformations that would interfere with diabetes treatment (e.g. congenital heart malformations, lung diseases, renal malformations)
19. Growth hormone deficiency
20. Combined Hypopituitarism
21. Down Syndrome (high risk for comorbidity with coeliac disease, autoimmune thyroiditis)
22. Cancer under treatment
23. Current participation in other interventional clinical trials

7.2 Recruitment

The study will aim for 72 randomised subjects. Recruitment will target up to 80 subjects to allow for drop-outs. Participants will be recruited from paediatric diabetes outpatient clinics at each of the following sites.

1. Addenbrooke's Hospital, Cambridge University Hospital NHS Foundation Trust, Cambridge, UK
2. Leeds Teaching Hospitals NHS Trust, Leeds, UK

Fuchs J, *et al. BMJ Open* 2021; 11:e042790. doi: 10.1136/bmjopen-2020-042790

3. Centre hospitalier de Luxembourg, Luxembourg
4. University of Leipzig, Leipzig, Germany
5. Medical University of Graz, Graz, Austria
6. Medical University of Innsbruck, Innsbruck, Austria
7. Medical University of Vienna, Vienna, Austria

Each centre will aim to recruit between 8 and 12 participants. Paediatric Diabetes Centres in the East Anglia region and London may be included as a Patient Identification Centre (PIC) to recruit participants for the Addenbrooke's site, and a centre in York will be a PIC for the Leeds site.

Potential participants will be identified by their treating clinicians and invited to contact the research team. They will be sent the study information leaflets and an invitation to join the study by the research team.

7.3 Randomisation

Eligible subjects will be randomised using central randomisation software. The randomisation will be stratified by centre.

8 Methods under Investigation

8.1 Name and Description of the Method of Investigation

The investigational treatment is hybrid closed-loop system, see section 4.5, or follow up prototypes of the automated closed loop insulin delivery system manufactured by the Cambridge University Hospitals NHS Foundation Trust. Component versions will be identified during regulatory submission to the national regulatory bodies.

8.2 Intended Purpose

The investigated medical device is used to manage glucose levels in people with type 1 diabetes, aged 1 year and older, using a hybrid closed loop approach.

8.3 Method of Administration

The closed loop system consists of components directly attached to the patient, which are the CGM sensor/transmitter and the insulin pump. The component not directly attached to the patient is the handheld smartphone containing closed loop algorithm and communicating wirelessly with the insulin pump.

8.4 Required Training

Prior to commencement of the study, the research team nurses/clinicians at each of the investigation centres will be trained to use the closed loop system and its components. Prior to the use of study devices, participants and parents/guardians will be trained to use the real-time CGM device and study pump, and the closed loop system. If participants and parents/guardians are already proficient in use of the study devices, training may be modified as deemed appropriate by the research team. Carers at nursery or school may be trained by the study team if required. Competency assessments of the family's capability to use study devices including the closed loop system will be made.

8.5 Precautions

During treatment with insulin there is a risk of hypoglycaemia and hyperglycaemia. In-hospital testing and hazard analysis have documented reduced risk of hypoglycaemia and hyperglycaemia during closed loop compared to conventional treatment.

8.6 Accountability of the Method under Investigation

The local Investigator will provide training for the study participants and will make every effort, through regular contact, to ascertain that the closed loop system is used for the study purposes only. Devices will be identified using batch/lot/serial numbers and the location of investigational devices and their dates of use by subjects will be documented throughout the study.

9 Study Schedule

9.1 Overview

The study will be coordinated from the Institute of Metabolic Science, Addenbrooke's Hospital, Cambridge, UK. The study will be performed at clinical sites in Austria, Germany, Luxembourg and UK.

After recruitment, consent, training and run-in period, subjects will be randomised to 4 months home use of real-time CGM combined with automated day and night closed loop insulin delivery or 4 months home use of sensor augmented pump therapy. Subjects will then crossover to the other study arm for the following 4 months. The order of the two interventions will be allocated at random. There will be a one to four week washout period between the two study periods.

The study will consist of up to 8 visits and 11 telephone/email contacts for subjects completing the study. The visit to set up automated closed loop for the first time may take place in the home setting or alternatively in a clinical facility. All other visits can take place at the hospital clinic, home or other suitable meeting place, according to participants' convenience. Maximum time in the study is 11 months.

Prior to the final visit all parents/guardians will be invited to participate in a sleep sub-study.

Table 8 outlines study activities when closed loop intervention precedes sensor augmented pump therapy.

Table 9 outlines study activities when sensor augmented pump therapy precedes closed loop intervention.

Fuchs J, *et al. BMJ Open* 2021; 11:e042790. doi: 10.1136/bmjopen-2020-042790

Table 8 Schedule of study visits when closed loop intervention precedes sensor augmented pump therapy

	Visit / contact	Description	Start relative to previous / next Visit / Activity	Duration	Comment
**Consent and training**	**Visit 1**	Recruitment visit: Consent/assent, HbA1c questionnaires	-	1-2 hours	
	**Visit 2**	CGM start: CGM training, initiation of CGM, competency assessment	Within 2 weeks of Visit 1 (may coincide with Visit 1)	2-3 hours	May be repeated if competency not achieved
	**Visit 3**	Pump start: insulin pump training, study pump initiation, competency assessment	Within 1 week of Visit 2 (may coincide with Visit 2)	2-4 hours	May be repeated if competency not achieved
**Run-in 2-4 weeks**	Contact 1	Review pump settings and CGM data; adjustment of treatment	After 1 week of visit 3 (± 3 days)	30 minutes	
	**Visit 4***	End of run-in, adjustment of treatment, treatment adherence assessment	After 2-4 weeks of visit 3 (minimum of 2 weeks)	1 hour	Run-in and Visit 4 may be repeated if non-compliant
		**Randomisation**	Immediately after Visit 4		If compliant with pump & CGM use
**CL Intervention 16 weeks**	**Visit 5**	CL initiation: training (CL), competency assessment	Within 1 week of Visit 4	1-2 hours	
	Contact 2	Follow up after CL start	Within 24 to 48 hours after Visit 5	30 minutes	
	Contact 3	Follow up, review use of study devices	After 1 week of Visit 5 (± 3 days)	30 minutes	
	Contact 4	Follow up, review use of study devices	After 4 weeks of Visit 5 (± 2 weeks)	30 minutes	
	Contact 5	Follow up, review use of study devices	After 8 weeks of Visit 5 (± 2 weeks)	30 minutes	
	Contact 6	Follow up, review use of study devices	After 12 weeks of Visit 5 (± 2 weeks)	30 minutes	
	**Visit 6**	End of 1st study arm: device download, HbA1c, questionnaires, interview	After 16 weeks of Visit 5 (± 2 weeks)	< 1 hour	
**WASHOUT PERIOD (1-4 WEEKS)**					
**Sensor augmented pump therapy 16 weeks**	**Visit 7**	Sensor augmented pump therapy initiation: refresher training	Within 1 week of Visit 6 (may coincide with Visit 6)	1-2 hours	
	Contact 7	Follow up after Sensor augmented pump therapy start	within 24 to 48 hours after Visit 7	30 minutes	
	Contact 8	Follow up, review use of study devices	After 1 week of Visit 7 (± 3 days)	30 minutes	
	Contact 9	Follow up, review use of study devices	After 4 weeks of Visit 7 (± 2 weeks)	30 minutes	
	Contact 10	Follow up, review use of study devices	After 8 weeks of Visit 7 (± 2 weeks)	30 minutes	
	Contact 11	Follow up, review use of study devices	After 12 weeks of Visit 7 (± 2 weeks)	30 minutes	

Fuchs J, *et al. BMJ Open* 2021; 11:e042790. doi: 10.1136/bmjopen-2020-042790

	**Visit 8 (caregiver only)**	Sleep assessment	After 12 weeks of Visit 7 (± 2 weeks) (Visit 8 may coincide with Contact 11)	30 minutes	
	**Visit 9**	End of study, return and download of devices, HbA1c, questionnaires, interview, resume standard pump therapy	After 16 weeks of Visit 7 (± 2 weeks)	<1 hour	
* could be done via phone/email					

Table 9 Schedule of study visits when sensor augmented pump therapy precedes closed loop intervention.

	Visit / contact	Description	Start relative to previous / next Visit / Activity	Duration	Comment
**Consent and training**	**Visit 1**	Recruitment visit: Consent/assent, HbA1c, questionnaires	-	1-2 hours	
	**Visit 2**	CGM start: CGM training, initiation of CGM, competency assessment	Within 2 weeks of Visit 1 (may coincide with Visit 1)	2-3 hours	May be repeated if competency not achieved
	**Visit 3**	Pump start: insulin pump training, study pump initiation, competency assessment	Within 1 week of Visit 2 (may coincide with Visit 2)	2-4 hours	May be repeated if competency not achieved
**Run-in 2-4 weeks**	Contact 1	Review pump settings and CGM data; adjustment of treatment	After 1 week of visit 3 (± 3 days)	30 minutes	
	**Visit 4***	End of run-in, adjustment of treatment, treatment adherence assessment	After 2-4 weeks of visit 3 (minimum of 2 weeks)	1 hour	Run-in and Visit 4 may be repeated if non-compliant
		**Randomisation**	Immediately after Visit 4		If compliant with pump & CGM use
**Sensor augmented pump therapy 16 weeks**	**Visit 5**	Sensor augmented pump therapy initiation: refresher training	Within 1 week of Visit 4	1-2 hours	
	Contact 2	Follow up after Sensor augmented pump therapy start	Within 24 to 48 hours after Visit 5	30 minutes	
	Contact 3	Follow up, review use of study devices	After 1 week of Visit 5 (± 3 days)	30 minutes	
	Contact 4	Follow up, review use of study devices	After 4 weeks of Visit 5 (± 2 weeks)	30 minutes	
	Contact 5	Follow up, review use of study devices	After 8 weeks of Visit 5 (± 2 weeks)	30 minutes	
	Contact 6	Follow up, review use of study devices	After 12 weeks of Visit 5 (± 2 weeks)	30 minutes	
	**Visit 6**	End of 1st study arm: device download, HbA1c, questionnaires, interview	After 16 weeks of Visit 5 (± 2 weeks)	<1 hour	
	**WASHOUT PERIOD (1-4 WEEKS)**				
**CL Interv ention 16**	**Visit 7**	CL initiation: training (CL), competency assessment	Within 1 week of Visit 6 (may coincide with Visit 6)	1-2 hours	

Fuchs J, *et al. BMJ Open* 2021; 11:e042790. doi: 10.1136/bmjopen-2020-042790

	Contact 7	Follow up after CL start	within 24 to 48 hours after Visit 7	30 minutes	
	Contact 8	Follow up, review use of study devices	After 1 week of Visit 7 (± 3 days)	30 minutes	
	Contact 9	Follow up, review use of study devices	After 4 weeks of Visit 7 (± 2 weeks)	30 minutes	
	Contact 10	Follow up, review use of study devices	After 8 weeks of Visit 7 (± 2 weeks)	30 minutes	
	Contact 11	Follow up, review use of study devices	After 12 weeks of Visit 7 (± 2 weeks)	30 minutes	
	**Visit 8 (caregiver only)**	Sleep assessment	After 12 weeks of Visit 7 (± 2 weeks) (Visit 8 may coincide with Contact 11)	30 minutes	
	**Visit 9**	End of study, return and download of devices, HbA1c, questionnaires, interview, resume standard pump therapy	After 16 weeks of Visit 7 (± 2 weeks)	<1 hour	
* could be done via phone/email					

9.2 Recruitment Visit (Visit 1)

During the screening visit the responsible investigator will inform the patient and the parents/guardians about the nature of the study and will answer all questions arising. If the family decides to participate in the study, the parents or legal representatives will be asked to sign the informed consent form before study-specific procedures are initiated. Whenever possible and according to local laws and recommendations of the local Ethics Committees, the assent of the subjects will be obtained in addition to the consent of the parents or legal representatives. All families will receive a copy of the informed consent/assent form.

During this visit, patients will be screened for eligibility according to the inclusion/exclusion criteria. Eligible subjects will have a blood test to determine their HbA1c. HbA1c eligibility criterion will be based on local measurement. Height and weight will be recorded. Parents/guardians will complete a set of questionnaires (see section 12.3) assessing participants' and families' quality of life, psychosocial functioning, diabetes management and response to their current treatment before entering the study. Visits 1-3 may be combined.

9.3 Study CGM Training (Visit 2)

The training session will cover key aspects of the study real-time CGM and particular attention will be paid to the following areas:

- Insertion and initiation of sensor session
- When a finger-stick BG reading needs to be taken
- How to calibrate successfully
- Blood glucose targets and alarm settings
- Handling real-time CGM feedback including glucose trend arrows, reported high and low glucose
- Use of software to upload and analyse CGM data

Written easy to use guidelines for the operation of study CGM will be provided. Sensor settings will be established. Parents/guardians will have to complete a competency assessment at the end of the training session. This training session could be repeated if competency was not achieved. Visits 1-3 may be combined.

9.4 Study Pump Training (Visit 3)

This session will cover key aspects of insulin pump use with respect to the study pump, and particular attention will be paid to the following areas:

- Importance of carbohydrate counting and refresher on carbohydrate counting skills
- Understanding insulin to carb ratios and correction factors
- Correct use of bolus calculator – subjects and parents/guardians will be required to use this bolus calculator for all insulin boluses during the study period
- Insulin cartridge and Infusion set changes and correct priming procedure
- Dealing with exercise
- Sick day rules
- Dealing with hypo- and hyperglycaemia
- Uploading data from study devices

Written easy to use guidelines for the operation of the study insulin pump will be provided. Parents/guardians will have to complete a competency assessment at the end of the training session. This session will be conducted by a professional pump educator ± member of the study team following a written curriculum. Those competent in the use of the study pump will be switched to the study pump. Training session could be repeated if competency is not achieved.

For self-monitoring of blood glucose (SMBG) throughout the study, subjects will continue using their own glucose meter provided the meter type meets ISO standards

Fuchs J, *et al. BMJ Open* 2021; 11:e042790. doi: 10.1136/bmjopen-2020-042790

(Standard 15197: 2013) and has the capability to allow study staff to download data at each visit. Whenever possible, the participant should use the same blood glucose meter throughout the study. If the subject's current meter is not compliant, a study meter will be provided for use. Appropriate devices and test strips will be provided at Visit 2 as required. Visits 1-3 may be combined.

9.5 Run-in

The family will use the study insulin pump and the study CGM over a 2 to 4-week run-in period. Pump, CGM and glucometer data will be downloaded from home on a regular basis. To download study devices, Glooko/Diasend software or similar will be used.

One week after Visit 3, parents/guardians will be contacted by email or telephone to assess device use, and to troubleshoot any problems. Uploaded data may be used for treatment optimisations. Additional contacts will be scheduled as required. Parents/guardians will also be able to contact the research team in between visits and contacts for support or to provide any additional training on the devices as required. Additional contacts and/or visits will be recorded on the study case report forms.

9.6 End of Run-in, Compliance Assessment (Visit 4)

At the end of the run-in, the families will be invited to attend the research centre or contacted via e-mail/telephone. Study insulin pump and CGM device will be downloaded and reviewed. Uploaded data may be used for treatment optimisations. There should be a minimum of two weeks run-in period for all subjects (end of Visit 3 to end of Visit 4).

During Visit 4, participant's compliance of using the study CGM and study pump over run-in will be assessed. To proceed with the study, subjects/guardians need to demonstrate correct use of study insulin pump and CGM including use of bolus calculator over 75% of meal boluses and at least 8 days' worth of CGM data during the last 14 days of the run-in period. Run-in could be repeated if compliance assessment was unsatisfactory. However, if subject/guardians repeatedly fail to demonstrate compliance, the study will be terminated and subject will be removed from the study.

9.7 Randomisation

Immediately after Visit 4, eligible subjects who have gained confidence in the use of the study insulin pump and the study CGM system, as assessed by the research team, will be randomised using a randomisation based on a computer-generated random code. Randomisation will take place centrally, with an independent person being responsible for the randomisation sequence. Subjects will be assigned to receive four months of day and night closed loop insulin delivery followed by four months of sensor augmented pump therapy, or vice versa.

9.8 Initiation of Study Arm Visits (Visit 5, Visit 7)

9.8.1 Closed Loop Insulin Delivery

Those subjects/guardians randomised to closed loop intervention will receive training required for safe and effective use of the closed loop system. This will include training on connection and disconnection of the closed loop system, and switching between closed loop and sensor augmented pump therapy. Written step by step guidance will also be provided. During the training session, subjects/guardians will use closed loop system under supervision from study staff. Competency on the use of closed loop system will be assessed by the study team. Only subjects/guardians who demonstrate competency on use of the system will be allowed to continue to the home study phase, and will be allowed to use closed loop at home without supervision by study staff.

Subjects/guardians are expected to use the closed loop at all times during the four month intervention period. Subjects/guardians are expected to upload study devices at regular intervals using Glooko/Diasend software or similar.

9.8.2 Sensor augmented pump therapy

Those subjects/guardians randomised to sensor augmented pump therapy will receive refresher training including key aspects of insulin pump and CGM use. During the 4-month treatment period, subjects/guardians will use the study pump and the study CGM, and will be followed up as outlined below.

Subjects/guardians are expected to upload study devices at regular intervals using Glooko/Diasend software or similar.

9.8.3 Remote Monitoring

During both study arms, in the event of hypo- or hyperglycaemia (levels predefined) a message may be sent to the parent/guardian providing real-time alerts. Parents/guardians will also be able to access sensor glucose readings and trends via Glooko/Diasend (or similar) software on-demand.

9.9 Telephone/Mail Contacts after Starting Study Treatment (Contacts 2-11)

During each of the study arms, the families will be contacted via telephone/e-mail approximately 24-48 hours (Contact 2, Contact 7), and 1 week after study arm initiation (Contact 3, Contact 8). The purpose of these contacts will be to review the use of study devices, to troubleshoot, or to provide any additional training required.

During both study periods, the families will be contacted via telephone/e-mail every month (Contacts 4-6, Contact 9-11). The purpose of these contacts will be to troubleshoot any problems, and to record any adverse events, device deficiencies, and changes in insulin settings, other medical conditions and/or medication.

After randomisation, subjects/parents and/or the clinical team are free to adjust insulin therapy as per usual clinical practice, but no active treatment optimisation will be undertaken by the research team.

9.10 End of 1st Study Arm (Visit 6)

Sixteen weeks after the start of the first treatment arm (Visit 5), participants will complete their respective study arm. Study devices will be downloaded. A blood sample will be taken for measurement of HbA1c (see section 12.2.1). Body weight and height measurements will be made. Parents/carers will be asked to complete questionnaires (see section 12.3). A subset of parents/carers will be invited to participate in an interview study (see section 12.4).

Subjects will then cross over to the other treatment arm. Those randomised to sensor augmented pump therapy initially will undergo closed loop training exactly as described above (see section 9.8.1). Those completing the closed loop arm will receive refresher training on key aspects of sensor augmented pump use exactly as described above (see section 9.8.2).

9.11 Washout

A one to four week washout period will follow when subjects/guardians could continue using the study pump and CGM (but not closed loop), or revert to their own insulin pump. Subjects will then cross over to the alternative intervention.

9.12 Visit 8

Parents/guardians will be invited to participate in a sleep sub-study prior to the final visit. Parents will be fitted with an Actiwatch (a simple wristwatch used to measure sleep non-invasively in the participant's home). The wristwatch will be worn for up to 7 days. Concomitantly, a sleep diary will be kept. PSQI and CSHQ questionnaires will be completed by the parents/guardians. The wristwatch, the completed diary and questionnaires, will be will be sent back to the research team or collected by the research team.

9.13 End of 2nd Study Arm (Visit 9)

Sixteen weeks after the start of the second treatment arm (Visit 7) subjects will have completed the study. Study devices will be downloaded. A blood sample will be taken for measurement of HbA1c (see section 12.2.1). Body weight and height measurements will be made. Parents/carers will be asked to complete questionnaires (see section 12.3). Parents/carers who were interviewed at the end of the 1st arm of the study will be re-interviewed (see section 12.4).

This will be the end of the study visit and subjects will return to their normal diabetes care. Subjects/guardians will be asked to return the study devices used, and will revert to their conventional insulin therapy by switching back to the insulin pump they were using before entering the study.

9.14 Participant Withdrawal Criteria

The following pre-randomisation withdrawal criteria will apply:

1. Subject/guardian is unable to demonstrate safe use of study insulin pump and CGM as judged by the investigator
2. Subject/guardian fails to demonstrate compliance with insulin pump and CGM during run-in

The following pre- and post-randomisation withdrawal criteria will apply:

3. Subjects/guardians may terminate participation in the study at any time without necessarily giving a reason and without any personal disadvantage
4. Significant protocol violation or non-compliance
5. Any severe hypoglycaemia event related to the use of the closed loop system

6. Two severe hypoglycaemia events not related to the use of the closed loop system
7. DKA unrelated to infusion site failure and related to the use of the closed loop system
8. Decision by the investigator or the sponsor that termination is in the subject's best medical interest
9. Allergic reaction to insulin
10. Allergic reaction to adhesive surface of infusion set or glucose sensor

Efforts will be made to retain subjects in follow up for the final primary endpoint assessment even if the intervention is discontinued, unless the investigator believes that it will be harmful for the subject to continue in the trial. Subjects/guardians who are withdrawn for reasons stated in (4) to (10) will be invited to provide a blood sample at the end of the planned study intervention for the assessment of HbA1c. Subjects/guardians who discontinue study intervention prior to the final visit will receive an exit survey and may be invited for an interview.

9.15 Study stopping criteria

The study may be stopped if three consecutive participants withdraw on safety grounds or on the advice of an independent Data Safety Monitoring Board (DSMB).

9.16 Co-enrolment Guidelines

To avoid potentially confounding issues, ideally participants should not be recruited into other trials. Where recruitment into another study is considered to be appropriate and without having any detrimental effect on the present study, this must first be discussed with the Chief Investigator, or the Sponsor or its representative.

Germany only
Participants will not be recruited into other trials and patients currently participating in other interventional clinical trials will be excluded from participation in KidsAP02.

9.17 Support Telephone Line

There will be a 24-hour telephone helpline to the local research teams for subjects/guardians in case of any technical device or problems related to diabetes management such as hypo- or hyperglycaemia. The local research team will have access to central 24 hour advice on technical issues.

Fuchs J, *et al. BMJ Open* 2021; 11:e042790. doi: 10.1136/bmjopen-2020-042790

9.18 Subject Reimbursement

The study will provide the CGM device, insulin pump, closed loop components and related consumables. Glucose meters and glucose test strips will also be provided where required. A study payment will be made to reflect local practice. The amount paid will be specified in the participant information sheet/informed consent form and REC application form. Reasonable travel expenses will also be reimbursed. After completing the study, subjects/guardians will not keep the study devices. They will revert to their conventional insulin pump therapy.

10 Endpoints

10.1 Primary Endpoints

The primary endpoint is the between group difference in time spent with sensor glucose levels between 3.9 to 10.0 mmol/l (70 to 180 mg/dl) during the 4 months intervention period.

10.2 Other Key Endpoints

- Time spent above target glucose (10.0 mmol/l) (180 mg/dl)
- HbA1c
- Average of glucose levels
- Time spent below target glucose (3.0 mmol/l) (70 mg/dl)

10.3 Secondary Endpoints

Secondary endpoints include:

- Standard deviation, and coefficient of variation of glucose levels
- Time with glucose levels <3.0 mmol/l (54 mg/dl)
- Time with glucose levels in significant hyperglycaemia (glucose levels > 16.7 mmol/l) (300 mg/dl)
- AUC of glucose below 3.5 mmol/l (63 mg/dl)
- BMI SDS
- Total, basal, and bolus insulin dose

Endpoints regarding glucose levels will be based on sensor glucose data.

Fuchs J, *et al. BMJ Open* 2021; 11:e042790. doi: 10.1136/bmjopen-2020-042790

10.4 Safety Evaluation

Safety evaluation will comprise the number of episodes of severe hypoglycaemia as well as the number of subjects experiencing severe hypoglycaemia, frequency of diabetic ketoacidosis, and other adverse events or serious adverse events.

All subjects including those who withdraw will be included in the safety evaluation.

10.5 Utility Evaluation

Utility evaluation is the frequency and duration of CGM use and use of the closed loop system at home.

- Percentage of time of closed loop operation
- Percentage of time of CGM availability

10.6 Human Factors

Human Factors evaluation will assess the emotional and behavioural characteristics of participating subjects and family members and their response to the closed-loop system and clinical trial using validated surveys, sleep assessment and semi-structured interviews.

10.7 Health Economics

Health economic analysis will be performed contrasting the artificial pancreas (closed-loop) and sensor augmented pump therapy using a health economic simulation model: the IMS IQ VIA Core Diabetes Model (CDM). Long-term outcomes derived from the simulation will include total direct costs, life expectancy, quality-adjusted life expectancy and time to onset of complications. Incremental costs versus incremental effectiveness (quality-adjusted life years [QALYs]) for closed-loop vs sensor augmented pump therapy will be compared.

11 Assessment and Reporting of Adverse Events

11.1 Definitions

Fuchs J, *et al. BMJ Open* 2021; 11:e042790. doi: 10.1136/bmjopen-2020-042790

Reportable Adverse Events

A reportable Adverse Event is any untoward medical occurrence that meets criteria for a serious adverse event. Device deficiencies that could have led to a serious adverse device effect will also be reported.

11.1.1 Adverse Events

An adverse event (AE) is any untoward medical occurrence, unintended disease or injury, or untoward clinical signs (including abnormal laboratory findings) in a subject who has received an investigational device, whether or not related to the
11.1.2 investigational medical device. This definition includes events related to the device under investigation or the comparator or to the study procedures. For users or other persons, this definition is restricted to events related to the investigational device.

The following anticipated adverse events will not be recorded:

- Non clinically significant skin reactions as judged by investigator
- Pre-existing medical conditions
- New illnesses or conditions not requiring concomitant medication or medical intervention/procedures
- Non severe hypoglycaemia
- Hyperglycaemia without significant ketonaemia (>0.6 mmol/l)

11.1.3 Adverse Device Effect

An Adverse Device Effect (ADE) is an adverse event related to the use of an investigational medical device. This includes adverse events resulting from insufficient or inadequate instructions for use, deployment, implantation, installation, or operation, or any malfunction of the investigational medical device. This definition also includes any event resulting from use error or from intentional misuse of the device under investigation.

The following anticipated adverse device effects will not be recorded:

11.1.4
- Non clinically significant skin reactions due to sensor or infusion set use as judged by investigator

Serious Adverse Event

A serious adverse event (SAE) is an adverse event that:

- led to a death
- led to a serious deterioration in the health of the subject, that either resulted in:
    - a life threatening illness or injury
    - a permanent impairment of a body structure or function
    - in-patient hospitalisation or prolonged hospitalisation

Fuchs J, *et al. BMJ Open* 2021; 11:e042790. doi: 10.1136/bmjopen-2020-042790

  - medical or surgical intervention to prevent life-threatening illness or injury or permanent impairment to a body structure or a body function
- led to foetal distress, foetal death or a congenital abnormality or birth defect

A planned hospitalisation for pre-existing condition, or a procedure required by the study protocol, without a serious deterioration in health, is not considered to be a serious adverse event.

More than one of the above criteria can be applicable to one event. Life-threatening in the definition of a serious adverse event or serious adverse reaction refers to an event in which the subject was at risk of death at the time of the event; it does not refer to an event which hypothetically might have caused death if it were more severe. Medical judgement should be exercised in deciding whether an adverse event or reaction is serious in other situations.

Important adverse events or reactions that are not immediately life-threatening or do not result in death or hospitalisation but may jeopardise the subject or may require intervention to prevent one of the other outcomes listed in the definition above, should also be considered serious.

The following serious adverse events, should they occur, will be classified as anticipated:

- Severe hypoglycaemia
- DKA

11.1.5 Serious Adverse Device Effect

A Serious Adverse Device Effect (SADE) is an adverse device effect that has resulted in any of the consequences characteristic of a serious adverse event.

11.1.6 Unanticipated Serious Adverse Device Effect

An Unanticipated Serious Adverse Device Effect (USADE) is a serious adverse device effect which by its nature, incidence, severity or outcome has not been identified in the current version of the protocol.

This includes unanticipated procedure related serious adverse events; that is, serious adverse events occurring during the study procedure that are unrelated to any malfunction or misuse of the investigational medical device.

Fuchs J, *et al. BMJ Open* 2021; 11:e042790. doi: 10.1136/bmjopen-2020-042790

An Anticipated Serious Adverse Device Effect (ASADE) is a serious adverse device effect which by its nature, incidence, severity or outcome has been identified in the protocol.

11.1.7 Device Deficiencies

A device deficiency is an inadequacy of a medical device with respect to its identity, quality, durability, reliability, safety or performance. A device deficiency may lead to an Adverse Device Effect or Serious Adverse Device Effect. The following anticipated device deficiencies and device-related issues will not be recorded:

- Infusion set occlusion/leakage not leading to ketonaemia
- Sensor failure due to miscalibration/detachment
- Premature interruption of sensor-life
- Battery lifespan deficiency due to inadequate charging or extensive wireless communication
- Control algorithm device error messages not needing system replacement
- Intermittent device communication failure not leading to system replacement

11.1.8 Adverse Event Intensity

Intensity	Definition
Mild	Patient is aware of signs and symptoms but they are easily tolerated
Moderate	Signs / symptoms cause sufficient discomfort to interfere with usual activities
Severe	Patient is incapable of working or performing usual activities

NB. The term “severe” is often used to describe the intensity (severity) of a specific event. This is not the same as ‘serious’, which is based on patient/event outcome or action criteria (see definition 11.1.4). For example, itching for several days may be rated as severe, but may not be clinically serious.

11.1.9 Adverse Event Causality

Intensity	Definition

Fuchs J, et al. BMJ Open 2021; 11:e042790. doi: 10.1136/bmjopen-2020-042790

Not assessable	A report suggesting an adverse event, which cannot be judged because information is insufficient or contradictory, and which cannot be supplemented or verified.
Unlikely	A clinical event, including laboratory test abnormality, with a temporal relationship, which makes a causal relationship improbable, and in which other drugs/treatments, chemicals or underlying disease(s) provide plausible explanations.
Possible	A clinical event, including laboratory test abnormality, with a reasonable time sequence to administration of the treatment/use of investigational treatment/device, but which also could be explained by concomitant diseases or other drugs/treatments or chemicals.
Probable	A clinical event, including laboratory test abnormality, with a reasonable time sequence to administration of the treatment/use of medical method/device, unlikely to be attributable to concomitant disease(s) or other drugs/treatments or chemicals, and which follows a clinically reasonable response on withdrawal (dechallenge). Rechallenge information is not required to fulfil this definition.
Definite/certain	A clinical event, including laboratory test abnormality, occurring in a plausible time relationship to study treatment/use of medical method/device and which cannot be explained by concomitant disease(s), other drugs/treatments or chemicals. The response to withdrawal of the treatment (dechallenge) should be clinically plausible. The event must be unambiguous, either pharmacologically or as phenomenon, using satisfactory rechallenge procedures if necessary.

(Reference: WHO-UMC Causality Categories)

11.2 Recording and Reporting of Adverse Events, Serious Adverse Events and Device Deficiencies

11.2.1 Monitoring Period of Adverse Events

The period during which adverse events will be reported is defined as the period from the beginning of the study (obtaining informed consent) until 3 weeks after the end of

the study participation. Adverse events that continue after the subject's discontinuation or completion of the study will be followed until their medical outcome is determined or until no further change in the condition is expected. The follow up of AEs may therefore extend after the end of the clinical investigation; however no new AEs will be reported after the trial reporting period.

11.2.2 Recording and Reporting of Adverse Events

Throughout the course of the study, all efforts will be made to remain alert to possible adverse events or untoward findings. The first concern will be the safety of the subject, and appropriate medical intervention will be taken. The investigator will elicit reports of adverse events from the subject at each visit and complete adverse event forms. All AEs, including those the subject/guardian reports spontaneously, those the investigators observe, and those the subject/guardian reports in response to questions will be recorded on paper or electronic AE forms at each site within seven days of discovering the event.

The study investigator will assess the relationship of any adverse event to be device-related or unrelated by determining if there is a reasonable possibility that the adverse event may have been caused by the study device or study procedures. The individual investigator at each site will be responsible for managing all adverse events according to local protocols, and decide if reporting is required.

11.2.3 Severe Hypoglycaemia

In line with current ISPAD consensus guidelines (59), hypoglycaemic events will be considered severe if the event requires assistance of another person due to altered consciousness to actively administer carbohydrate, glucagon, or other resuscitative actions. This means that the subject is impaired cognitively to the point that he/she is unable to treat him- or herself, is unable to verbalize his or her needs, is incoherent, disoriented, and/or combative, or experiences seizure or coma. If plasma glucose measurements are not available during such an event, neurological recovery attributable to the restoration of plasma glucose to normal is considered sufficient evidence that the event was induced by a low plasma glucose concentration.

Severe hypoglycaemia will be regarded as a foreseeable serious adverse event and a serious adverse event form will be completed. Non-severe hypoglycaemia will not be reported or considered an adverse event.

11.2.4 Diabetic Ketoacidosis

Biochemical criteria for the diagnosis of ***diabetic ketoacidosis (DKA)*** are defined as per current ISPAD consensus guidelines (60):

Fuchs J, et al. BMJ Open 2021; 11:e042790. doi: 10.1136/bmjopen-2020-042790

- hyperglycaemia (blood glucose >200 mg/dl or >11 mmol/l)
- with either low pH (<7.3) or low serum bicarbonate (<15 mmol/l)
- and ketonemia or ketonuria

DKA will be regarded as a foreseeable serious adverse event and a serious adverse event form will be completed.

11.25 Reporting of Serious Adverse Events and Serious Adverse Device Effects

When reporting adverse events, all pertinent data protection legislation must be adhered to.

The serious adverse event report should contain the following information*:

1. Study identifier (EudraCT number if applicable)
2. Participant's unique study number
3. Date of birth
4. Event description
5. Start date of event
6. Laboratory tests used and medical interventions used to treat the SAE
7. Planned actions relating to the event, including whether the study device was discontinued
8. Statement on the patient's current state of health
9. Reason for seriousness (i.e. death, life threatening, hospitalisation, disability/incapacity or other)
10. Evaluation of causality (including grade of relatedness) with the following (more than one may apply):
    a. the investigational treatment/medical device
    b. the clinical study/a study specific procedure
    c. other: e. g. concomitant treatment, underlying disease
11. Reporter's name, date and signature

*In the case of incomplete information at the time of initial reporting, all appropriate information should be provided as soon as this becomes available.

The relationship of the SAE to the investigational treatment / medical device should be assessed by the investigator at site, as should the anticipated or unanticipated nature of any SAEs and SADEs.

Fuchs J, *et al. BMJ Open* 2021; 11:e042790. doi: 10.1136/bmjopen-2020-042790

All SAEs whether or not deemed investigational method/device related and whether anticipated or unanticipated must be reported to the Sponsor by email or fax within 24 hours (one working day) of the Investigator learning of its occurrence.

SAEs should be reported to:
Stephen Kelleher
Cambridge University Hospitals
NHS Foundation Trust
Box 277, Addenbrooke's Hospital
Hills Road, Cambridge, CB2 0QQ, UK
Phone: +44 (0) 1223 217418
Fax: +44 (0) 1223 348494
E-mail: enquiries@addenbrookes.nhs.uk.

A written report must follow within five working days and is to include a full description of the event and sequelae, in the format detailed on the Serious Adverse Event reporting form. If applicable, the Sponsor will notify the competent authority of all Serious Adverse Events in line with pertinent legal requirements.

The Investigator will notify the Research Ethics Committee (REC) in UK of all Serious Adverse Events in line with pertinent legal requirements. The Investigator will inform the Sponsor about all reports sent to the reporting organisation including follow-up information and answers by the reporting organisation. The local investigator is responsible for informing other site principal investigators and the CI of all SAEs.

The respective national regulatory authority (e.g. MHRA) will be notified of all SAEs as soon as possible within ten days of the event occurring during the study. The main REC will be notified of all unexpected and related SAEs within 15 days of the occurrence of the event.

11.2.6 Recording and Reporting of Device Deficiencies

All device deficiencies will be documented throughout the study. The investigator at each site will be responsible for managing all device deficiencies and determine and document in writing whether they could have led to a serious adverse device effect.

All device deficiencies that might have led to a serious adverse device effect(s) if: suitable action had not been taken, intervention had not been made, or if circumstances had been less fortunate, must be reported to the Sponsor as for SAEs/SADEs.

Fuchs J, et al. BMJ Open 2021; 11:e042790. doi: 10.1136/bmjopen-2020-042790

11.2.7 Healthcare Arrangements and Compensation for Adverse Events

Healthcare arrangements for subjects who suffer an adverse event as a result of participating in the study may include advice from clinical members of the study team or the patient's treating diabetes team, or use of emergency health services.

If an adverse event occurs, there are no special compensation arrangements unless this was due to the negligence of one of the clinical investigators or due to harm resulting from study protocol design. In this case subjects may have grounds for legal action for compensation. The normal national complaints mechanism will be available. In addition, any harm arising due to study design (both negligent and non-negligent) will be covered under Sponsor's insurance policy as applicable.

11.2.8 Country specific requirements

1. UK - The Investigator will notify the ethics committee of all Serious Adverse Events in line with pertinent legal requirements. The Investigator will inform the Sponsor about all reports sent to the ethics committee including follow-up information and answers by the ethics committee. The MHRA and REC will be notified of all SAEs/SADEs occurring during the study according to the timelines specified in section 11.1.5.

2. Germany - According to the German Ordinance on Medical Devices Vigilance (Medizinprodukte-Sicherheitsplanverordnung – MPSV), §2 (5) a Serious Adverse Event (SAE) is defined as:

   „Ein schwerwiegendes unerwünschtes Ereignis ist jedes in einer genehmigungspflichtigen klinischen Prüfung oder einer genehmigungspflichtigen Leistungsbewertungsprüfung auftretende ungewollte Ereignis, das unmittelbar oder mittelbar zum Tod oder zu einer schwerwiegenden Verschlechterung des Gesundheitszustands eines Probanden, eines Anwenders oder einer anderen Person geführt hat, geführt haben könnte oder führen könnte ohne zu berücksichtigen, ob das Ereignis vom Medizinprodukt verursacht wurde; das Vorgesagte gilt entsprechend für schwerwiegende unerwünschte Ereignisse, die in einer klinischen Prüfung oder Leistungsbewertungsprüfung, für die eine Befreiung von der Genehmigungspflicht nach § 20 Absatz 1 Satz 2 des Medizinproduktegesetzes erteilt wurde, aufgetreten sind.“

Fuchs J, *et al. BMJ Open* 2021; 11:e042790. doi: 10.1136/bmjopen-2020-042790

(English translation: A serious adverse event in a licensable clinical trial or a licensable performance evaluation is an unintended event which directly or indirectly caused, may have caused in the past, or may cause in the future, death or a serious aggravation of the state of health of a patient, a user or another person, without considering whether or not the event was caused by the medical device; this applies also to serious adverse events, which occur in a clinical trial or performance evaluation, for which an exemption of approval was granted according to § 20 paragraph 1 sentence 2 of the German Medical Devices Act)

According to MPG and MPGSV §5:
a) The sponsor has to report all SAEs with possible causal relationship to the investigational medical device (=SADE) and all SAEs with possible causal relationship to study procedure to BfArM immediately (BfArM SAE form).
b) The sponsor has to report SAEs without possible causal relationship to the investigational medical device or procedure to BfArM quarterly (MEDDEVform and as report) or upon request.

3. Austria - All SAEs have to be documented by the sponsor and immediately reported according to § 42 (8) and § 70 of the Austrian Medical Device Directive (StF: BGBl. Nr. 657/1996, BGBl. I Nr. 143/2009) to the competent authority (AGES) and the competent authorities of other countries within the European Union where the study is conducted. All SAEs must be reported using the templates provided by AGES. The Investigator must notify the affected ethics committee without delay of any serious side effects and any serious adverse events during the clinical trial (MPG § 61). The clinical investigator must inform the sponsor of any medical device effects and any serious adverse events during the clinical trial (MPG § 64, (5)). Furthermore a continuous reporting form (tabular listing = line listing) F_I287 (SAE report table) must be maintained for all SAEs (occurring abroad or in Austria), and the BASG must immediately be notified if new SAEs or changes to or additions to previously reported SAEs occurred.

4. Luxembourg - The Investigator will notify the ethical committee (CNER) of all Serious Adverse Events in line with pertinent legal requirements (directives 90/385/eec and 93/42/eec). The Investigator will inform the Sponsor about all reports sent to the ethics committee including follow-up information and answers by the ethics committee. The Competent Authority will be notified of all SAEs/SADEs occurring during the study according to the timelines specified in section 11.2.5.

11.3 Anticipated Adverse Events, Risks and Benefits

11.3.1 Risks and Anticipated Adverse Events

Known risks represent hazardous situations which may result in anticipated adverse events. In the following text, where appropriate, the term “risk” and “anticipated adverse events” are used interchangeably without affecting meaning.

11.3.2 Hypoglycaemia and Hyperglycaemia

Subjects with type 1 diabetes have a pre-existing risk of hypoglycaemia and hyperglycaemia. Potential risks are:

- Risk of mild to moderate hypoglycaemia and associated symptoms such as sweating, trembling, difficulty thinking and dizziness. There is also a rare risk of severe hypoglycaemia when conscious level is altered, needing help from a third party to correct the hypoglycaemia. These risks are pre-existent in any patient with type 1 diabetes and the study objective is to develop systems to minimise these risks
- Risk of possible mild to moderate hyperglycaemia similar to the risk that a subject with type 1 diabetes experiences on a daily basis
- Risk of hyperglycaemia leading to diabetic ketoacidosis (DKA). This risk is pre-existent in any patient with type 1 diabetes.

11.3.3 Blood Sampling for HbA1c and Blood Glucose Measurements

Finger-prick tests may produce pain and/or bruising at the site.

11.3.4 Insulin Pump Therapy

Patients participating in this study are already using an insulin pump. Risks associated with insulin pump therapy include:

- Slight discomfort at the time of insertion of the insulin delivery cannula (common)
- Slight bruising at the site of insertion (common)
- Infusion set and cannula occlusions (rare)
- Bleeding at insertion site (rare)
- Infection at the site of insertion (rare)
- Allergy to the insulin delivery cannula or adhesive (rare)
- Insulin pump malfunction and mechanical problems (rare)
- Allergy to insulin (very rare)

Fuchs J, *et al. BMJ Open* 2021; 11:e042790. doi: 10.1136/bmjopen-2020-042790

- Lipodystrophy / lipoatrophy (very rare)

Continuous Glucose Monitoring

Risks associated with CGM include:

- Slight discomfort at the time of insertion of CGM (common)
- 11.3.5 Slight bruising at the site of insertion (unlikely)
- Bleeding at insertion site (rare)
- Infection at the site of insertion (rare)
- Allergic reaction to the CGM sensor material (rare)

If a skin reaction is classified as severe (the observation is noticeable and bothersome to subject/guardian and may indicate infection or risk of infection or potentially life-threatening allergic reaction), an adverse event form will be completed.

Questionnaires and Interviews

11.3.6 As part of the study, parents/guardians will complete questionnaires and take part in interviews which include questions about their private attitudes, feelings and behaviour related to diabetes. It is possible that some people may find the questions to be mildly upsetting. Similar questionnaires and interview structures have been used in previous research and these reactions are uncommon. If questionnaire responses or interview discussions indicate serious psychological distress as judged by the investigators, appropriate clinical services will be arranged. Any treatment will be documented in the case-report form.

The study teams take the safeguarding of children very seriously and should any concerns be raised during the course of the study, including questionnaires and interviews, these concerns will be dealt with in accordance with local policy. Parents/guardians will be made aware of this.

11.3.7

Risk Analysis and Residual Risk Associated with the Investigational Device

After in-depth analysis and consideration of all the potential hazards in relation to use of the FlorenceX system in the home environment, it is concluded that the FlorenceX system is safe, if used as intended.

Risk Assessment of the FlorenceX system has been carried out in accordance with ISO 14971:2012. A preliminary Hazard Determination has been carried out including consideration of the questions in Annex C of ISO 14971:2012.

One hazard 'Hazard S7: Incorrect Calibration of Blood Glucose Sensor due to error of reading resulting in overestimation of blood glucose' is the only hazard identified that could not be reduced to an acceptable risk level, post mitigation. Our in-detail risk/benefit assessment concluded that the benefits of the system outweigh the risk with respect to this specific hazard.

As per our risk management process, further risk analysis shall be undertaken post production and release as to ensure any issues raised are acted upon to ensure the FlorenceX system continues to improve and develop.

11.4 Benefits

It is expected that day and night closed loop may have an important role in the management of diabetes. The closed loop system may impact on the frequency of hypoglycaemia with suspected fewer low glucose levels with closed loop insulin delivery compared with sensor augmented pump therapy. The closed loop system may lead to an improvement in quality of life and reduction of parental fears regarding hypoglycaemia in very young children with type 1 diabetes. In addition to this, higher blood glucose levels above target should be reduced with use of the closed loop algorithm. Therefore, participation in this study is likely to lead to improved metabolic control and be beneficial for study participants.

It is possible that subjects will not directly benefit from being a part of this study. However, it is also possible that the blood glucose information from the CGM device along with the information about insulin dosing during day and night closed loop will be useful for subjects' diabetes self-management.

Further information on benefits - Germany only

It is expected that day and night closed loop may have an important role in the management of diabetes. The closed loop system will have impact on frequency of hypoglycemia with suspected fewer low glucose levels in the closed loop setting compared to regular treatment. In addition to this also higher blood glucose levels beyond the target should be reduced with the used algorithm. Especially in the night blood glucose levels closer to target should be reached. It is expected that within the study, metabolic control will be better controlled in when using closed loop. Therefore, the results of this study are likely to be beneficial for subjects with diabetes. The study may have an impact on the long term outcome of the study participants.

Fuchs J, *et al. BMJ Open* 2021; 11:e042790. doi: 10.1136/bmjopen-2020-042790

The implementation of a closed loop system with improved near target blood glucose levels and reduced rate of hypoglycemia will then lead to improvement in quality of life, metabolic control and reduction of parental fears regarding hypoglycemia and outcome of very young children with type 1 diabetes.

It is possible that subjects will not directly benefit from being a part of this study. However, it is also possible that the blood sugar information from the CGM devices along with the information about insulin dosing during day and night closed loop will be useful for subjects' diabetes self-management.

11.5 Data Safety Monitoring Board (DSMB)

An independent Data Safety Monitoring Board (DSMB) will be informed of all serious adverse events and any unanticipated adverse device effects that occur during the study and will review compiled adverse event data at periodic intervals.

12 Methods and Assessments

12.1 Procedures

12.1.1 Height and Weight

These will be recorded at the study initiation visit at baseline and at the end of each study arm. Height will be measured in centimetres using calibrated measuring devices. Weight will be measured in kilograms using a calibrated electronic scale.

12.1.2 Blood Glucose Meter Data

The blood glucose meter will be downloaded periodically during the whole duration of the study.

12.1.3 Insulin Pump and CGM Data

The study pump and study CGM data will be downloaded periodically during the control arm by the participant and during study visits.

12.2 Laboratory Methods

12.2.1 HbA1c

A blood sample for the measurement of HbA1c levels will be taken at three different time points: baseline and at the end of each study arm. HbA1c will be measured using an International Federation of Clinical Chemistry and Laboratory Medicine (IFCC)

aligned method. All HbA1c testing must follow National Glycohemoglobin Standardization Program (NGSP) standards.

Total Blood Loss

The total blood loss will be approximately 300 µl.

12.2.112.3 Questionnaires

Quantitative data on health-related quality of life will be assessed using validated questionnaires. Parents/guardians will complete a series of validated questionnaires (**Error! Reference source not found.**) at baseline and at the end of each study arm. I t is estimated that parents/guardians will take 15-25 minutes to complete the surveys. All results will be evaluated at the end of the study. All questionnaires are available in English, French and German

Table 10 – Questionnaires to be completed by parents/guardians at baseline and at the end of each intervention arm (Visit 1, 6, and 8).

Measure	***Construct Measured / Relevant Points***	***Duration***
**Strengths and Difficulties** Questionnaire (SDQ) (61)	This is a widely used 25 item self-report inventory behavioural screening questionnaire for children and adolescents. The same 25 items are included in questionnaires for completion by the parents.	5-10 min
**Hypoglycaemia Fear** Survey (HFS) Parents of Young Children (62-64)	Validated questionnaire to measure several dimensions of fear of hypoglycaemia. It consists of a “Behaviour subscale” that measures behaviours involved in avoidance and over-treatment of hypoglycaemia and a “Worry subscale” that measures anxiety and fear surrounding hypoglycaemia.	5- 10 min
**WHO Well-Being Index** (WHO-5) (65)	Widely used 5-item questionnaire assessing subjective psychological well-being.	2-3 min
**Epworth sleepiness** scale (ESS) (66)	Simple 8-item questionnaire to measure the parents general level of daytime sleepiness and average sleep propensity in daily life.	3-5 min
Insulin delivery Systems: Perceptions, Ideas, Reflections, and	Measures the psychological side of automated insulin delivery. Child version has 27 items; Parent version has 30 items.	5-10 min

Fuchs J, *et al. BMJ Open* 2021; 11:e042790. doi: 10.1136/bmjopen-2020-042790

Expectations **(INSPIRE) Survey**		
Pittsburgh Sleep Quality Index (PSQI)	The PSQI is a 19 item questionnaire that holistically assesses sleep quality and sleep duration.	5-10 min
Children's Sleep Habit Questionnaire (CSHQ)	The CSHQ is a validated retrospective 45-item questionnaire that examines sleep behaviour in young children.	10-15 min

At the end of the closed loop intervention arm, parents'/guardians' experience using closed loop will be documented using the parent closed loop experience questionnaire.

12.4 Sleep

Quality, duration and fragmentation of sleep will be assessed subjectively (using the Pittsburgh Sleep Quality Index (PSQI) or similar validated questionnaire, and a daily sleep diary) and objectively (by actigraphy) in parents/guardians. These measures will be conducted over 7 days at Visit 8 prior to the end of the study in both arms.

The PSQI is a validated 19 item questionnaire that holistically assesses sleep quality and sleep duration. The CSHQ is a validated 45 item questionnaire examining sleep behaviour in young children. The sleep diary will record time of going to bed and waking, plus time of, and reason for any nocturnal awakenings.

An Actiwatch (Philips Respironics, Bend, Oregon, USA) worn on the non-dominant wrist will provide objective measures of sleep and wakefulness based on motor activity - a low cost, non-invasive and objective method for evaluating sleep in free-living participants. Actiwatches will record time in bed and actual sleep time, as well as changes in sleep quality from measures of sleep maintenance, sleep efficiency, sleep latency, fragmentation index, total nocturnal activity, and percentage moving time. Light exposure will be measured by the Actiwatch's photovoltaic sensor.

12.5 Qualitative Interview

A subset of parents/guardians will be interviewed at the end of each study arm. Approximately 30 parents/guardians will be interviewed with representation from each

Fuchs J, *et al. BMJ Open* 2021; 11:e042790. doi: 10.1136/bmjopen-2020-042790

study country. Parents will be asked to opt-in to the interview study when they enter the study.

The objective of the interviews is to understand and explore parents' experiences of using closed loop insulin delivery compared with sensor-augmented pump therapy to manage their child's diabetes. The same participants/guardians will be interviewed after completing each study arm to look at whether, in what ways, and why, use of a closed loop system (as compared to sensor-augmented pump therapy) has impacted on their diabetes management practices, their worries and concerns about hyperglycaemia and hypoglycaemia; and their work and family lives. Interviews will also explore participants' likes and dislikes of using the closed loop system and how the technology might be improved for future use. Interviews will be informed by topic guides which will enable the discussion to stay relevant to the study aims while affording the flexibility needed for participants to discuss issues they see as important, including those unforeseen at the outset of the study.

Interviews will take place at a time chosen by the parents/guardians and will be carried out by telephone or Skype (where local regulations permit). Participants will be interviewed in English or German by an experienced qualitative researcher who is fluent in both languages. Interviews will be audio-recorded with consent. All interviews will be transcribed in full for in-depth analysis; with interviews undertaken in German translated into English.

12.6 Health Economics

The analysis will be performed using the IQVIA Core Diabetes Model (CDM; QVIA, Basel, Switzerland). The CDM is a validated non-product-specific policy analysis tool for cost-effectiveness analysis in both type 1 and type 2 diabetes; a detailed description of the model architecture (including schematic diagrams) and validation is available in publications by Palmer et al (67, 68) and more recently McEwan et al (69). In summary, the model is based on a series of inter-dependent submodels that simulate both acute and long-term diabetes-related complications (angina, myocardial infarction, congestive heart failure, stroke, peripheral vascular disease, diabetic retinopathy, macula oedema, cataract, hypoglycaemia, ketoacidosis, lactic acidosis, depression, oedema, nephropathy and end-stage renal disease, neuropathy, foot ulcer and amputation, and non-specific mortality). The sub-models have a semi-Markov structure and use time, state, time-in-state and diabetes type-dependent probabilities derived from published sources to simulate disease progression. Monte Carlo simulation using tracker variables is used to overcome the memory-less

Fuchs J, *et al. BMJ Open* 2021; 11:e042790. doi: 10.1136/bmjopen-2020-042790

properties of the standard Markov model and allows for interconnectivity and interaction between individual sub-models.

Simulation cohort and treatment effects

Baseline characteristics of the simulation cohort will come from the study. They will include: age, sex ratio, Hb1Ac and other risk factors. Treatment effects will be based on the study findings comparing 4 months of closed-loop with 4 months of sensor
12.6.1 augmented pump therapy.

Costs and utilities

Country specific direct costs will be sourced from published literature and where
12.6.2 necessary inflated to the current year costs.

For treatment costs, only the incremental costs between the two arms will be considered, namely the difference between closed-loop therapy and sensor augmented pump therapy.

Health state utility values will be taken from published literature (70) and references therein.

13 Study Materials and Products

13.1 Insulin

The participant’s pre-enrolment rapid or ultra-rapid acting insulin analogue (U-100 100U/ml or at a concentration used pre-enrolment) will be used in the insulin pumps during run-in, washout and during both study arms. Diluted insulin is a standard treatment approach for children with low insulin requirements. Study participants using diluted insulin prior to study enrolment will remain on diluted insulin throughout the study.

13.2 Insulin Pump

During the run-in period and both study arms, the Dana insulin pump (SOOIL) will be used. Glooko/Diasend software or similar will be used to download insulin pump data at regular intervals.

13.3 Continuous Subcutaneous Glucose Monitor

Fuchs J, *et al. BMJ Open* 2021; 11:e042790. doi: 10.1136/bmjopen-2020-042790

The Dexcom G6 real-time sensor with sensor applicator (Dexcom, Northridge, CA, USA) will be the study CGM. The sensor will be calibrated according to manufacturer's instructions.

13.4 Smartphone

An Android smartphone hosting FlorenceX App (see **Error! Reference source not found.**) will be used.

13.5 Blood Glucose Meter

Study participants will use their own approved glucose meter for self-monitoring of capillary blood glucose (SMBG) during the study. The capillary glucose meter readings may be used to calibrate the sensor according to manufacturer's instructions.

13.6 Computer-Based Algorithm

The Cambridge closed loop controller has been used safely and effectively in the closed loop studies in both children and adults with T1D (study REC Ref. 06/Q0108/350, REC Ref. 07/H0306/116, REC Ref. 08/H0304/75, REC Ref. 08/H0308/297, REC Ref. 09/H0306/44, REC Ref. 10/H0304/87, REC Ref. 12/EE/0155, REC Ref. 12/EE/0034, REC Ref. 12/EE/0424, REC Ref. 13/EE/0120, REC Ref. 13/WM/0498, REC Ref. 13/EE/0251, REC Ref. 13/EE/0321, REC Ref. 13/EE/0018, REC Red 15/EE/0324, REC ref 16/EE/0286, REC ref 16/EE/0380 and REC Ref 17/LO/0576).

13.7 Actiwatch

An Actiwatch (Philips Respironics, Bend, Oregon, USA) will be used to measure sleep over 7 day at Visit 8 prior to the end of the study in both arms.

14 Data Analysis

14.1 Primary Endpoint Analysis

The primary analysis will evaluate the between group difference in time spent in the target glucose range from 3.9 to 10.0 mmol/l (70 to 180 mg/dl) based on CGM glucose levels during the four month intervention periods.

Mean ± SD or summary statistics appropriate to the distribution will be reported for the primary endpoint over the four month period by treatment intervention. The treatment interventions will be compared using a linear mixed model adjusting for period as a fixed effect and site as a random effect. The model will account for correlated data from the same subject. A 95% confidence interval will be reported for the difference between the interventions based on the linear mixed model.

Residual values will be examined for an approximate normal distribution. If values are highly skewed, then a ranked normal score transformation will be used instead. However, previous experience suggests that the primary endpoint will follow an approximately normal distribution. A separate model will also be built with the inclusion of a period by treatment interaction to assess for the presence of a carryover effect. A two-sided p-value will be reported.

The primary analysis will be a single statistical comparison of a single outcome measure. No formal corrections for multiple comparisons will be performed for the secondary analyses in this study.

The primary analysis will be performed on an intention-to-treat basis using the treatment order assigned by randomization. Only subjects with the minimum number of hours of CGM data as specified in the analysis plan will be included.

A per-protocol analysis restricted to participants with a minimum of 60% CGM data during control period and 60% use of closed-loop during closed-loop period will be conducted for the primary endpoint.

A detailed analysis plan will be provided separately.

Primary endpoint hypotheses

- *Null Hypothesis:* There is no difference in the true mean time spent in the target range (3.9 to 10.0 mmol/L) over the four month period between the two treatment groups.
- *Alternative Hypothesis:* There is a nonzero difference in the true mean time spent in the target range over the four month period between the two treatment groups.

14.2 Other Key Endpoints

For the following key endpoints, the familywise type I error rate (FWER) will be controlled at two sided α = 0.05. A gatekeeping strategy will be used, where the

Fuchs J, *et al. BMJ Open* 2021; 11:e042790. doi: 10.1136/bmjopen-2020-042790

primary endpoint will be tested first, if passing the significance testing, other key endpoints will be tested in the order listed below using the fixed-sequence method at $\alpha = 0.05$.

- Time spent with sensor glucose levels between 3.9 to 10.0 mmol/l (70 to 180 mg/dl)
- Time spent above target glucose (10.0 mmol/l) (180 mg/dl)
- HbA1c
- Average of glucose levels
- Time spent below target glucose (3.9 mmol/l) (70 mg/dl)

14.3 Secondary Efficacy Analyses

The following endpoints will be considered exploratory and Benjamini-Hochberg false discovery rate (FDR) adjusted p-values will be calculated within each subcategory below:

CGM derived indices:

- Standard deviation, and coefficient of variation of glucose levels
- Time with glucose levels <3.0 mmol/l (54 mg/dl)
- Time with glucose levels in significant hyperglycaemia (glucose levels > 16.7 mmol/l) (300 mg/dl)
- AUC of glucose below 3.5 mmol/l (63 mg/dl)

Insulin and Other Endpoints:

- BMI SDS
- Total, basal, and bolus insulin dose

For all secondary endpoints, summary statistics appropriate to the distribution will be tabulated by treatment group. Analysis of all secondary CGM and insulin endpoints will parallel the primary analysis. The models comparing BMI standard deviation score and HbA1c will also adjust for baseline value at the start of each period.

For BMI standard deviation score and HbA1c, a longitudinal model adjusting for period as a fixed effect will be constructed to compare treatment arms. The model will include two time points: (1) period 1 outcome, and (2) period 2 outcome.

A ranked normal score transformation will be applied to all highly skewed secondary endpoints.

Fuchs J, *et al. BMJ Open* 2021; 11:e042790. doi: 10.1136/bmjopen-2020-042790

Summary statistics for the following outcome metrics will also be tabulated separately for daytime (defined as 8am to less than 12am) and night time (defined as 12am to less than 8am) over the four month period:

- Percent time with glucose levels spent in the target range (3.9 to 10.0 mmol/L)
- Mean of glucose levels
- Standard deviation of glucose levels
- Percent time with glucose levels below 3.9 mmol/L
- Total insulin dose

14.4 Safety Analyses

For each of the following safety outcomes, mean ± SD or summary statistics appropriate to the distribution will be tabulated by treatment group:

- Number of subjects with any diabetic ketoacidosis events
- Number of episodes of diabetic ketoacidosis events per subject and incidence rate per 100 person years
- Number of subjects with any severe hypoglycaemia events
- Number of episodes of severe hypoglycaemia events per subject and incidence rate per 100 person years
- Number of adverse events per subject
- Number of serious adverse events per subject

For purposes of analysis, a severe hypoglycaemic event will be defined as described in section 11.2.3.

All of the above safety outcomes will be tabulated for all subjects (including dropouts and withdrawals), regardless of whether CGM data are available and irrespective of whether closed loop was operational. All adverse events will be listed for the entire study duration, including run-in and washout period.

For each of diabetic ketoacidosis and severe hypoglycaemia (if enough events), the event rates will be compared using a repeated measures Poisson regression model adjusting for period. Binary variables will also be compared using a repeated measures logistic regression model adjusting for period.

14.5 Utility Evaluation

The amount of CGM use will be tabulated for each treatment arm, in addition to the amount of closed loop system use in the CL arm. Summary statistics appropriate to the distribution and range will be reported for the percentage of time using the CGM

Fuchs J, *et al. BMJ Open* 2021; 11:e042790. doi: 10.1136/bmjopen-2020-042790

over the four month period (as defined above) for each treatment group. The same will be done for the percentage of time using the closed loop system in the CL arm. Tabulations of summary statistics will also be performed for the percentage of time spent using the closed loop system while using the CGM in the CL arm.

14.6 Questionnaires

For each questionnaire (and their corresponding subscales), total scores will be calculated and reported at each time point. They will also be compared between treatment arms using the same model described above for the primary endpoint. The distribution of responses for each individual question at each time point will also be reported in a separate table.

14.7 Sleep assessment

Sleep will be automatically scored by Actiware software using previously described and validated algorithms. Sleep duration will be calculated as the sum of all epochs scored as sleep during the time in bed. Variability across nights in a parent/guardian's sleep duration will be summarised using the coefficient of variation.

14.8 Interviews

To maximise rigour at least two experienced qualitative researchers will be involved in data analysis. A thematic analysis will be undertaken by these individuals who will independently review data and write separate reports before attending regular meetings to compare their interpretations and reach agreement on recurrent themes and findings. Interviews conducted in German will be translated and transcribed into English prior to data analysis.

Interviews will be read through repeatedly and cross-compared in order to identify issues and themes which cut across different participant's accounts.

A key aspect of the analysis will involve comparison of the experiences and views of participants in the sensor augmented pump therapy arm and closed loop arm of the study, to better understand the impact of closed loop as compared to sensor augmented pump therapy on diabetes self-management practices and quality of life.
A final coding frame, reflecting the initial research questions and emergent themes, will be developed once all data have been reviewed and consensus reached on key

Fuchs J, et al. BMJ Open 2021; 11:e042790. doi: 10.1136/bmjopen-2020-042790

themes and findings. NVivo9, a qualitative software package, will be used to facilitate data coding/retrieval.

14.9 Health Economics Evaluation

For each simulation, a simulated cohort of 1,000 patients will be run through the model 1,000 times using first-order Monte Carlo simulation. Long-term outcomes will include total direct costs, life expectancy, quality-adjusted life expectancy and time to onset of complications. Future costs and clinical benefits will be discounted based on each country recommendations. The mean values from the simulation (a total of 1,000 mean values, each from a cohort of 1,000 patients run through the model) will then be used to generate scatterplots of incremental costs versus incremental effectiveness (quality-adjusted life years [QALYs]) for closed loop vs. sensor augmented pump therapy. Data from the scatterplot will then be used to generate a cost-effectiveness acceptability curve.

Sensitivity analysis
In order to explore the robustness of the base-case findings and establish the key drivers of results, a series of one-way simulations will be performed on those parameters.

Additional exploratory health-economic analysis might be conducted on other endpoints such as but not limited to sleep disorders and indirect costs for the society.

14.10 Interim Analysis

No formal interim analyses or stopping guidelines are planned for this study.

14.11 Sample Size and Power Calculations

The study will run at seven sites. We have ensured adequate recruitment of participants and centres by allowing for a potential 50% uptake and 10% drop-out of participants among collaborative paediatric sites, with further contingency sites including participant identification sites (identified but not included as applicants).
Data from the SENCE study (NCT02912728) were used to estimate the standard deviation of the primary endpoint. Only subjects from the two treatment arms involving CGM use in this study who were using an insulin pump at the time of enrolment were used to estimate the SD. Based on data from this study, 65 subjects are required to attain 90% power to detect a difference if a treatment effect of 5%, a standard deviation

Fuchs J, *et al. BMJ Open* 2021; 11:e042790. doi: 10.1136/bmjopen-2020-042790

of 10.3% for an individual measurement, and a correlation of 0.3 between periods are assumed. Adding an additional 10% to this sample size to account for drop outs results in a final sample size of 72 subjects.

14.12 Deviations from the Statistical Plan

Any deviations from the original statistical plan will be recorded and agreed by the Investigators.

15 Case Report Forms

The Case Report Form (CRF) is the printed, optical, or electronic document designed to record all the protocol required information to be reported to the Chief Investigator for each study subject.

CRFs will be completed in accordance with GCP and ISO 14551 Guidelines. Corrections to the CRF will be performed by striking through the incorrect entry and by writing the correct value next to the data that has been crossed out; each correction will be initialled and explained (if necessary) by the Investigator or the Investigator's authorised staff.

The electronic CRF system provides an edit feature that records the identity of the person making the change and retains a record of the before and after values of the data field(s) in question. In addition, all eCRF changes require electronic review and sign-off by the investigator associated with the visit.

If any amendments to the protocol or other study documents are made, CRFs will be reviewed to determine if an amendment to these forms is also necessary.

16 Data Management

Confidentiality of subject data shall be observed at all times during the study. Personal details for each subject taking part in the research study and linking them to a unique identification number will be held locally on a study screening log in the Trial Master File at the investigation centre. These details will not be revealed at any other stage during the study, and all results will remain anonymous. The study identification number will be used on the case report forms and on all the blood and serum samples that are collected throughout the study. Names and addresses will not be used.

Collected samples will be stored securely and locked away. Only researchers directly involved in the study will have access to the samples.

Electronic data will be stored on password-protected computers. All paper records will be kept in locked filing cabinets, in a secure office at the investigation centre. Only members of the research team and collaborating institutions will have password access to the anonymised electronic data. Only members of the research teams will have access to the filing cabinet. Paper copies of the data will be stored for at least 15 years in line with the General Data Protection Regulation (GDPR) (EU) 2016/679.

Direct access to the source data will be provided for monitoring, audits, REC review and regulatory authority inspections during and after the study. The fully anonymised data may be shared with third parties (EU or non-EU based) for the purposes of advancing management and treatment of diabetes.

Appropriate procedures agreed by the Chief Investigator and Principal Clinical Investigator will be put in place for data review, database cleaning and issuing and resolving data queries.

17 Ethics

The study will be conducted in accordance with the Declaration of Helsinki Ethical Principles for Medical Research involving Human Subjects (64th WMA General Assembly, Fortaleza, Brazil, October 2013).

17.1 Independent Research Ethics Committees (REC)

Prior to commencement of the study, the protocol, any amendments, subject information and informed consent and assent forms, any other written information to be provided to the subject, subject recruitment procedures, current investigator CVs, and any other documents as required by the relevant Research Ethics Committee will be submitted. Written approval will be obtained from the relevant Ethics Committee prior to the commencement of the study. Any additional requirements imposed by the REC or regulatory authority shall be followed.

17.2 Informed consent of study subjects

In obtaining and documenting informed consent, the investigator will comply with the applicable regulatory requirements and will adhere to ICH GCP standards and to the

ethical principles that have their origin in the Declaration of Helsinki. Prior to the start of the study, the Investigator will obtain favourable ethical opinion of the written informed consent form, assent form and any other written information to be provided to subjects.

Subjects and parents/guardians will be given full verbal and written information regarding the objectives and procedures of the study and the possible risks involved. The study team will avoid any coercion or undue improper inducement of the subject to participate and subjects and parents/guardians will be given ample time to consider participation in the study.

If the family decides to participate in the study, the parents or legal representatives will be asked to sign the informed consent form before study-specific procedures are initiated. Whenever possible and according to local laws and recommendations of the local Ethics Committees, the assent of the subjects will be obtained in addition to the consent of the parents or legal representatives. All subjects will receive a copy of the informed consent/assent form, and the Project Coordinator's office will hold copies of the consent/assent forms and Ethics Committee approvals and make them available upon request.

All subjects will have the right to leave the study at any time, without stating any reason, and without any negative consequences to their subsequent medical treatment. The subject and/or their legal representative will be informed in a timely manner should any new information become available during the course of the study that may affect their well-being, safety and willingness to participate in the study.

18 Amendments to the Protocol

Any substantial amendments to the protocol and other documents shall be notified to, and approved by, the Research Ethics Committee, and the regulatory authority, prior to implementation as per nationally agreed guidelines.

19 Deviations from Protocol

Deviations from the protocol should not occur without prior approval of the REC or sponsor except under emergency circumstances, to protect the rights, safety and well-being of subjects. If deviations do occur, they will be documented, stating the reason and the date, the action taken, and the impact for the subject and for the study. The documentation will be kept in the Investigator's Site File. Deviations will be logged

Fuchs J, et al. BMJ Open 2021; 11:e042790. doi: 10.1136/bmjopen-2020-042790

electronically and will require chief investigator or local principal investigator acknowledgement and sign-off.

Deviations affecting the subject's rights, safety and well-being or the scientific integrity of the study will be reported to the REC and sponsor as soon as possible in a timely manner, following nationally-agreed guidelines.

20 Study Management

20.1 Data and Safety Monitoring Board (DSMB)

An independent Data Safety Monitoring Board (DSMB) will comprise an independent chairperson and two external experts. The DSMB aims to safeguard the interests of trial participants, assess the safety and efficacy of the interventions during the trial, and monitor the overall conduct of the clinical trial.

The DSMB should receive and review the progress and accruing data of the project clinical trials and provide advice on the conduct of the trial. The DSMB will be informed of all serious adverse events and any unanticipated adverse device effects that occur during the study and will review compiled adverse event data at periodic intervals.

20.2 Trial Steering Committee (TSC)

The Trial Steering Committee (TSC) will provide the overall supervision of the clinical trial. The TSC will comprise an independent chairperson, the Chief Investigator, and the leaders of work packages (WP) 2 (pilot study), WP3 (main study) and WP4 (data management) of the KidsAP consortium. The TSC will monitor clinical trial progress and conduct and advise on scientific credibility. The TSC will consider and act, as appropriate, upon the recommendations of the Data Safety and Monitoring Board (DSMB) and ultimately carries the responsibility for deciding whether a trial needs to be stopped on grounds of safety or efficacy.

The TSC will meet (in person or conference call) at regular intervals during the preparation of the study and follow up to discuss the operational aspects of the study. The Principal Clinical Investigators may also participate. The TSC will report its decisions to the Ethical Board and the General Assembly of the KidsAP Consortium.

20.3 Study Monitoring

The Study Coordinator will ensure that the study is conducted in accordance with ICH GCP standards through site monitoring visits. A monitoring plan will be written and agreed prior to randomisation.

21 Responsibilities

21.1 Chief Investigator

The Chief Investigator (CI) is the person with overall responsibility for the research and all ethical applications will be submitted by the CI. The CI is accountable for the conduct of the study and will ensure that all study personnel are adequately qualified and informed about the protocol, any amendments to the protocol, the study treatments and procedures and their study related duties. The CI should maintain a list of appropriately qualified persons to whom he/she has delegated specified significant study-related duties.

21.2 Principal Clinical Investigator

The Principal Clinical Investigator will be responsible for the day-to-day conduct of the clinical aspects of the study.

21.3 Study Coordinator

The Study Coordinator will provide day-to-day support for the study and provide training through Principal Investigator meetings, site initiation and routine monitoring visits.

22 Reports and Publications

Data will be submitted for publication in internationally peer-reviewed scientific journals; members of the investigator group will all be co-authors. The privacy of each subject and confidentiality of their information shall be preserved in reports and publication of data.

23 Timetable

Inclusion of the first subject in the study is planned to take place in January 2019. The expected completion of the last subject is March 2020 and the planned completion of the Clinical Study Report is June 2020.

24 Retention of Study Documentation

Subject notes must be kept for the maximum time period as permitted by each relevant institution. Other source documents and the Investigator's Site File must be retained for at least 15 years, in line with the General Data Protection Regulation (GDPR) (EU) 2016/679. The Principal Investigator will archive the documentation pertaining to the study after completion or discontinuation of the study.

25 Indemnity Statements

Indemnity for any harm arising from the conduct of research will be provided according to local arrangements in respective centres.

- Cambridge, UK - National Health Service indemnity cover will apply for any claims arising from management and conduct of research. Any liability arising from study design will be covered by the clinical trial insurance policy organised by the University of Cambridge.
- Graz, Innsbruck, Vienna, Austria – Subjects will be insured according to Medical Device Law § 47 (StF: BGBl. Nr. 657/1996, BGBl. I Nr. 143/2009)
- Leipzig, Germany - Subjects will be insured according to the German Medical Device Law (MPG). Any liability arising from the study will be covered by the clinical trial insurance policy organized by the University of Leipzig.
- Luxembourg - Centre Hospitalier de Luxembourg indemnity will apply for any claims arising from the management and conduct of research. Any liability arising from the study design will be covered by the clinical trial insurance policy organised by the Centre Hospitalier de Luxembourg (Amlin Corporate Insurance NV).

Fuchs J, *et al. BMJ Open* 2021; 11:e042790. doi: 10.1136/bmjopen-2020-042790

26 References

1. Todd JA. Etiology of type 1 diabetes. Immunity. 2010;32(4):457-67.

2. Gale EA. The rise of childhood type 1 diabetes in the 20th century. Diabetes. 2002;51(12):3353-61.

3. Patterson CC, Gyurus E, Rosenbauer J, Cinek O, Neu A, Schober E, et al. Trends in childhood type 1 diabetes incidence in Europe during 1989-2008: evidence of non-uniformity over time in rates of increase. Diabetologia. 2012;55(8):2142-7.

4. Wood JR, Miller KM, Maahs DM, Beck RW, DiMeglio LA, Libman IM, et al. Most youth with type 1 diabetes in the T1D Exchange Clinic Registry do not meet American Diabetes Association or International Society for Pediatric and Adolescent Diabetes clinical guidelines. Diabetes Care. 2013;36(7):2035-7.

5. Maahs DM, Hermann JM, DuBose SN, Miller KM, Heidtmann B, DiMeglio LA, et al. Contrasting the clinical care and outcomes of 2,622 children with type 1 diabetes less than 6 years of age in the United States T1D Exchange and German/Austrian DPV registries. Diabetologia. 2014;57(8):1578-85.

6. Bohn B, Karges B, Vogel C, Otto K-P, Marg W, Hofer SE, et al. 20 Years of Pediatric Benchmarking in Germany and Austria: Age-Dependent Analysis of Longitudinal Follow-Up in 63,967 Children and Adolescents with Type 1 Diabetes. PLOS ONE. 2016;11(8):e0160971.

7. Juvenile Diabetes Research Foundation Continuous Glucose Monitoring Study G, Tamborlane WV, Beck RW, Bode BW, Buckingham B, Chase HP, et al. Continuous glucose monitoring and intensive treatment of type 1 diabetes. N Engl J Med. 2008;359(14):1464-76.

8. Tsalikian E, Fox L, Weinzimer S, Buckingham B, White NH, Beck R, et al. Feasibility of prolonged continuous glucose monitoring in toddlers with type 1 diabetes. Pediatr Diabetes. 2012;13(4):301-7.

9. Hovorka R. Closed-loop insulin delivery: from bench to clinical practice. Nature reviews Endocrinology. 2011;7(7):385-95.

10. Bakhtiani PA, Zhao LM, El Youssef J, Castle JR, Ward WK. A review of artificial pancreas technologies with an emphasis on bi-hormonal therapy. Diabetes Obes Metab. 2013.

11. Cobelli C, Renard E, Kovatchev B. Artificial pancreas: past, present, future. Diabetes. 2011;60(11):2672-82.

Fuchs J, *et al. BMJ Open* 2021; 11:e042790. doi: 10.1136/bmjopen-2020-042790

12. Hovorka R, Elleri D, Thabit H, Allen JM, Leelarathna L, El-Khairi R, et al. Overnight closed loop insulin delivery in young people with type 1 diabetes: A free-living randomised clinical trial. Diabetes Care. 2014;37(5):1204-11.

13. Thabit H, Lubina-Solomon A, Stadler M, Leelarathna L, Walkinshaw E, Pernet A, et al. Home use of closed-loop insulin delivery for overnight glucose control in adults with type 1 diabetes: a 4-week, multicentre, randomised crossover study. Lancet Diabetes Endocrinol. 2014;2(9):701-9.

14. Leelarathna L, Dellweg S, Mader JK, Allen JM, Benesch C, Doll W, et al. Day and night home closed-loop insulin delivery in adults with type 1 diabetes: three-center randomized crossover study. Diabetes Care. 2014;37(7):1931-7.

15. Tauschmann M, Allen JM, Wilinska ME, Thabit H, Stewart Z, Cheng P, et al. Day-and-night hybrid closed-loop insulin delivery in adolescents with type 1 diabetes: A free-living, randomized clinical trial. Diabetes Care. 2016;39(7):1168-74.

16. Thabit H, Tauschmann M, Allen JM, Leelarathna L, Hartnell S, Wilinska ME, et al. Home use of an artificial beta cell in type 1 diabetes. N Engl J Med. 2015;373(22):2129-40.

17. Kropff J, Del Favero S, Place J, Toffanin C, Visentin R, Monaro M, et al. 2 month evening and night closed-loop glucose control in patients with type 1 diabetes under free-living conditions: a randomised crossover trial. Lancet Diabetes Endocrinol. 2015;3(12):939-47.

18. Kovatchev BP, Renard E, Cobelli C, Zisser HC, Keith-Hynes P, Anderson SM, et al. Feasibility of outpatient fully integrated closed-loop control: first studies of wearable artificial pancreas. Diabetes Care. 2013;36(7):1851-8.

19. Del Favero S, Boscari F, Messori M, Rabbone I, Bonfanti R, Sabbion A, et al. Randomized Summer Camp Crossover Trial in 5- to 9-Year-Old Children: Outpatient Wearable Artificial Pancreas Is Feasible and Safe. Diabetes Care. 2016.

20. Russell SJ, Hillard MA, Balliro C, Magyar KL, Selagamsetty R, Sinha M, et al. Day and night glycaemic control with a bionic pancreas versus conventional insulin pump therapy in preadolescent children with type 1 diabetes: a randomised crossover trial. Lancet Diabetes Endocrinol. 2016;4(3):233-43.

21. Russell SJ, El-Khatib FH, Sinha M, Magyar KL, McKeon K, Goergen LG, et al. Outpatient glycemic control with a bionic pancreas in type 1 diabetes. N Engl J Med. 2014;371(4):313-25.

22. Ly TT, Roy A, Grosman B, Shin J, Campbell A, Monirabbasi S, et al. Day and Night Closed-Loop Control Using the Integrated Medtronic Hybrid Closed-Loop System in Type 1 Diabetes at Diabetes Camp. Diabetes Care. 2015;38(7):1205-11.

23. Nimri R, Danne T, Kordonouri O, Atlas E, Bratina N, Biester T, et al. The "Glucositter" overnight automated closed loop system for type 1 diabetes: a randomized crossover trial. Pediatr Diabetes. 2013;14(3):159-67.

24. Nimri R, Muller I, Atlas E, Miller S, Fogel A, Bratina N, et al. MD-Logic overnight control for 6 weeks of home use in patients with type 1 diabetes: randomized crossover trial. Diabetes Care. 2014;37(11):3025-32.

25. Phillip M, Battelino T, Atlas E, Kordonouri O, Bratina N, Miller S, et al. Nocturnal glucose control with an artificial pancreas at a diabetes camp. N Engl J Med. 2013;368(9):824-33.

26. de Beaufort CE, Swift PG, Skinner CT, Aanstoot HJ, Aman J, Cameron F, et al. Continuing stability of center differences in pediatric diabetes care: do advances in diabetes treatment improve outcome? The Hvidoere Study Group on Childhood Diabetes. Diabetes Care. 2007;30(9):2245-50.

27. Holl RW, Swift PG, Mortensen HB, Lynggaard H, Hougaard P, Aanstoot HJ, et al. Insulin injection regimens and metabolic control in an international survey of adolescents with type 1 diabetes over 3 years: results from the Hvidore study group. Eur J Pediatr. 2003;162(1):22-9.

28. Mauras N, Beck R, Xing D, Ruedy K, Buckingham B, Tansey M, et al. A randomized clinical trial to assess the efficacy and safety of real-time continuous glucose monitoring in the management of type 1 diabetes in young children aged 4 to <10 years. Diabetes Care. 2012;35(2):204-10.

29. Gaudieri PA, Chen R, Greer TF, Holmes CS. Cognitive function in children with type 1 diabetes: a meta-analysis. Diabetes Care. 2008;31(9):1892-7.

30. Ryan CM. Why is cognitive dysfunction associated with the development of diabetes early in life? The diathesis hypothesis. Pediatr Diabetes. 2006;7(5):289-97.

31. Ferguson SC, Blane A, Wardlaw J, Frier BM, Perros P, McCrimmon RJ, et al. Influence of an early-onset age of type 1 diabetes on cerebral structure and cognitive function. Diabetes Care. 2005;28(6):1431-7.

32. Schoenle EJ, Schoenle D, Molinari L, Largo RH. Impaired intellectual development in children with Type I diabetes: association with HbA(1c), age at diagnosis and sex. Diabetologia. 2002;45(1):108-14.

33. Persson S, Dahlquist G, Gerdtham UG, Steen Carlsson K. Impact of childhood-onset type 1 diabetes on schooling: a population-based register study. Diabetologia. 2013;56(6):1254-62.

34. Marzelli MJ, Mazaika PK, Barnea-Goraly N, Hershey T, Tsalikian E, Tamborlane W, et al. Neuroanatomical correlates of dysglycemia in young children with type 1 diabetes. Diabetes. 2014;63(1):343-53.

35. Barnea-Goraly N, Raman M, Mazaika P, Marzelli M, Hershey T, Weinzimer SA, et al. Alterations in White Matter Structure in Young Children With Type 1 Diabetes. Diabetes Care. 2013.

36. Williams RM, Dunger DB. Insulin treatment in children and adolescents. Acta Paediatr. 2004;93(4):440-6.

37. Bulsara MK, Holman CD, Davis EA, Jones TW. The impact of a decade of changing treatment on rates of severe hypoglycemia in a population-based cohort of children with type 1 diabetes. Diabetes Care. 2004;27(10):2293-8.

38. Murphy HR, Wadham C, Hassler-Hurst J, Rayman G, Skinner TC, Group FaACaTSF. Randomized trial of a diabetes self-management education and family teamwork intervention in adolescents with Type 1 diabetes. Diabet Med. 2012;29(8):e249-54.

39. Skyler JS. Struggles with clinical translation of immune intervention trials. Diabetes Care. 2014;37(5):1173-5.

40. Danne T, Kordonouri O, Holder M, Haberland H, Golembowski S, Remus K, et al. Prevention of hypoglycemia by using low glucose suspend function in sensor-augmented pump therapy. Diabetes technology & therapeutics. 2011;13(11):1129-34.

41. Choudhary P, Shin J, Wang Y, Evans ML, Hammond PJ, Kerr D, et al. Insulin pump therapy with automated insulin suspension in response to hypoglycemia: reduction in nocturnal hypoglycemia in those at greatest risk. Diabetes Care. 2011;34(9):2023-5.

42. Garg S, Brazg RL, Bailey TS, Buckingham BA, Slover RH, Klonoff DC, et al. Reduction in duration of hypoglycemia by automatic suspension of insulin delivery: the in-clinic ASPIRE study. Diabetes technology & therapeutics. 2012;14(3):205-9.

43. Bergenstal RM, Klonoff DC, Garg SK, Bode BW, Meredith M, Slover RH, et al. Threshold-based insulin-pump interruption for reduction of hypoglycemia. N Engl J Med. 2013;369(3):224-32.

44. Buckingham BA, Raghinaru D, Cameron F, Bequette BW, Chase HP, Maahs DM, et al. Predictive low-glucose insulin suspension reduces duration of nocturnal hypoglycemia in children without increasing ketosis. Diabetes Care. 2015;38(7):1197-204.

45. Guerci B, Meyer L, Salle A, Charrie A, Dousset B, Ziegler O, et al. Comparison of metabolic deterioration between insulin analog and regular insulin after a 5-hour interruption of a continuous subcutaneous insulin infusion in type 1 diabetic patients. The Journal of clinical endocrinology and metabolism. 1999;84(8):2673-8.

46. Ly TT, Nicholas JA, Retterath A, Davis EA, Jones TW. Analysis of glucose responses to automated insulin suspension with sensor-augmented pump therapy. Diabetes Care. 2012;35(7):1462-5.

47. Hovorka R, Allen JM, Elleri D, Chassin LJ, Harris J, Xing D, et al. Manual closed-loop insulin delivery in children and adolescents with type 1 diabetes: a phase 2 randomised crossover trial. Lancet. 2010;375(9716):743-51.

48. Elleri D, Allen JM, Kumareswaran K, Leelarathna L, Nodale M, Caldwell K, et al. Closed-loop basal insulin delivery over 36 hours in adolescents with type 1 diabetes: randomized clinical trial. Diabetes Care. 2013;36(4):838-44.

49. Nimri R, Muller I, Atlas E, Miller S, Kordonouri O, Bratina N, et al. Night glucose control with MD-Logic artificial pancreas in home setting: a single blind, randomized crossover trial-interim analysis. Pediatr Diabetes. 2014;15(2):91-9.

50. Barnard KD, Wysocki T, Allen JM, Elleri D, Thabit H, Leelarathna L, et al. Closing the loop overnight at home setting: psychosocial impact for adolescents with type 1 diabetes and their parents. BMJ Open Diabetes Res Care. 2014;2(1):e000025.

51. Elleri D, Allen JM, Tauschmann M, El-Khairi R, Benitez-Aguirre P, Acerini CL, et al. Feasibility of overnight closed-loop therapy in young children with type 1 diabetes aged 3-6 years: comparison between diluted and standard insulin strength. BMJ Open Diabetes Res Care. 2014;2(1):e000040.

52. Stewart ZA, Wilinska ME, Hartnell S, Temple RC, Rayman G, Stanley KP, et al. Closed-Loop Insulin Delivery during Pregnancy in Women with Type 1 Diabetes. N Engl J Med. 2016;375(7):644-54.

53. Tauschmann M, Allen JM, Wilinska ME, Thabit H, Acerini CL, Dunger DB, et al. Home use of day-and-night hybrid closed-loop insulin delivery in suboptimally controlled adolescents with type 1 diabetes: a 3-week, free-living, randomized crossover trial. Diabetes Care. 2016;39(11):2019-25.

54. Thabit H, Elleri D, Leelarathna L, Allen J, Lubina-Solomon A, Stadler M, et al. Unsupervised overnight closed loop insulin delivery during free living: analysis of randomised cross-over home studies in adults and adolescents with type 1 diabetes. Lancet. 2015;385 Suppl 1:S96.

55. Elleri D, Acerini CL, Allen JM, Hayes J, Pesterfield C, Wilinska ME, et al. Parental attitudes towards overnight closed-loop glucose control in children with type 1 diabetes. Diabetes technology & therapeutics. 2010;12(1):35-9.

56. Barnard KD, Wysocki T, Thabit H, Evans ML, Amiel S, Heller S, et al. Psychosocial aspects of closed- and open-loop insulin delivery: closing the loop in adults with Type 1 diabetes in the home setting. Diabet Med. 2015;32(5):601-8.

57. Hovorka R. Artificial Pancreas project at Cambridge 2013. Diabet Med. 2015;32(8):987-92.

58. Thabit H, Tauschmann M, Allen JM, Leelarathna L, Hartnell S, Wilinska ME, et al. Home use of an artificial beta cell in type 1 diabetes. N Engl J Med. 2015;373(22):2129-40.

59. Ly TT, Maahs DM, Rewers A, Dunger D, Oduwole A, Jones TW, et al. ISPAD Clinical Practice Consensus Guidelines 2014. Assessment and management of hypoglycemia in children and adolescents with diabetes. Pediatr Diabetes. 2014;15 Suppl 20:180-92.

60. Wolfsdorf JI, Allgrove J, Craig ME, Edge J, Glaser N, Jain V, et al. ISPAD Clinical Practice Consensus Guidelines 2014. Diabetic ketoacidosis and hyperglycemic hyperosmolar state. Pediatr Diabetes. 2014;15 Suppl 20:154-79.

61. Goodman R. The Strengths and Difficulties Questionnaire: a research note. J Child Psychol Psychiatry. 1997;38(5):581-6.

62. Green LB, Wysocki T, Reineck BM. Fear of hypoglycemia in children and adolescents with diabetes. J Pediatr Psychol. 1990;15(5):633-41.

63. Cox DJ, Irvine A, Gonder-Frederick L, Nowacek G, Butterfield J. Fear of hypoglycemia: quantification, validation, and utilization. Diabetes Care. 1987;10(5):617-21.

64. Gonder-Frederick LA, Schmidt KM, Vajda KA, Greear ML, Singh H, Shepard JA, et al. Psychometric properties of the hypoglycemia fear survey-ii for adults with type 1 diabetes. Diabetes Care. 2011;34(4):801-6.

65. Bonsignore M, Barkow K, Jessen F, Heun R. Validity of the five-item WHO Well-Being Index (WHO-5) in an elderly population. Eur Arch Psychiatry Clin Neurosci. 2001;251 Suppl 2:II27-31.

66. Johns MW. A new method for measuring daytime sleepiness: the Epworth sleepiness scale. Sleep. 1991;14(6):540-5.

67. Palmer AJ, Roze S, Valentine WJ, Minshall ME, Foos V, Lurati FM, et al. The CORE Diabetes Model: Projecting long-term clinical outcomes, costs and cost-

effectiveness of interventions in diabetes mellitus (types 1 and 2) to support clinical and reimbursement decision-making. Current medical research and opinion. 2004;20 Suppl 1:S5-26.

68. Palmer AJ, Roze S, Valentine WJ, Minshall ME, Foos V, Lurati FM, et al. Validation of the CORE Diabetes Model against epidemiological and clinical studies. Current medical research and opinion. 2004;20 Suppl 1:S27-40.

69. McEwan P, Foos V, Palmer JL, Lamotte M, Lloyd A, Grant D. Validation of the IMS CORE Diabetes Model. Value in health : the journal of the International Society for Pharmacoeconomics and Outcomes Research. 2014;17(6):714-24.

70. Beaudet A, Clegg J, Thuresson PO, Lloyd A, McEwan P. Review of utility values for economic modeling in type 2 diabetes. Value in health : the journal of the International Society for Pharmacoeconomics and Outcomes Research. 2014;17(4):462-70.

27 Document Amendment History

Version Number	Date	Amendment information
1.1	19/12/2018	1. Inclusion of additional exclusion criterion for Germany and UK only.
1.2	10/01/2019	1. Clarification of inclusion criteria<br>2. Section 13.4 “Smartphone” added for clarity<br>3. Clarification added on analysis of primary end point
1.3	17/07/2019	1. Change of clinical PI at Cambridge, UK
2.0	10/03/2020	1. Inclusion of sub-study on sleep assessment<br>2. Addition of 2 new questionnaires for caregivers
